# Phenotypic plasticity and cryptic generic enigma in *Pleosporales*: Establishing *Sporidesmioidesaceae* fam. nov. and *Rostriconidium synnematicum* sp. nov. from India

**DOI:** 10.3897/mycokeys.138.184414

**Published:** 2026-07-30

**Authors:** Sruthi O. Paraparath, Kunhiraman C. Rajeshkumar, Shadma Siddiqui, Dilna Chandran, Rungtiwa Phookamasak, Junfu Li, Nalin N. Wijayawardene, Rajnish Kumar Verma, Samantha C. Karunarathna, Sawoluck Tibpromma, Ajay Kumar Gautam, Rupam Kapoor, Nakarin Suwannarach, Rajesh Jeewon

**Affiliations:** 1 Biodiversity and Palaeobiology (Fungi) Gr., MACS Agharkar Research Institute, G. G. Agarkar Road, Pune, 411 004, Maharashtra, India School of Pharmacy, Zunyi Medical University Zunyi China; 2 Faculty of Science, Savitribai Phule Pune University, Pune, 411 007, Maharashtra, India Yunnan International Joint Laboratory of Fungal Sustainable Utilization in South and Southeast Asia, College of Biology and Food Engineering, Qujing Normal University Qujing China; 3 Department of Public Health, College of Applied Medical Sciences in Al-Namas, University of Bisha,255, Al-Namas City, Bisha 67398, Saudi Arabia Center for Yunnan Plateau Biological Resources Protection and Utilization, College of Biology and Food Engineering, Qujing Normal University Qujing China; 4 Department of Economic Plants and Biotechnology, Yunnan Key Laboratory for Wild Plant Resources, Kunming Institute of Botany, Chinese Academy of Sciences, Kunming, Yunnan 650201, China Honghe Center for Mountain Futures, Kunming Institute of Botany, Chinese Academy of Sciences Kunming China; 5 Honghe Center for Mountain Futures, Kunming Institute of Botany, Chinese Academy of Sciences, Honghe County 654400, Kunming, Yunnan, China Yunnan Key Laboratory for Wild Plant Resources, Kunming Institute of Botany, Chinese Academy of Sciences Kunming China; 6 Center for Yunnan Plateau Biological Resources Protection and Utilization, College of Biology and Food Engineering, Qujing Normal University, Qujing 655099, China College of Science, King Saud University Riyadh Saudi Arabia; 7 Yunnan International Joint Laboratory of Fungal Sustainable Utilization in South and Southeast Asia, College of Biology and Food Engineering, Qujing Normal University, Qujing 655099, China Department of Plant Pathology, Punjab Agricultural University Punjab India; 8 Department of Bioprocess Technology, Faculty of Technology, Rajarata University, Mihintale, Sri Lanka College of Applied Medical Sciences in Al-Namas, University of Bisha Bisha Saudi Arabia; 9 Tropical Microbiology Research Foundation, 96/N/10, Meemanagoda Road, Pannipitiya, 10230, Sri Lanka Faculty of Science, Savitribai Phule Pune University Pune India; 10 Mycology Lab, Department of Plant Pathology, Punjab Agricultural University, Ludhiana 141 004, Punjab, India Department of Botany, University of Delhi Delhi India; 11 Patanjali Herbal Research Department, Patanjali Research Institute, Haridwar 249405, Uttarakhand, India MACS Agharkar Research Institute Pune India; 12 Plant-Fungus Interactions Laboratory, Department of Botany, University of Delhi, Delhi 110007, India Center of Excellence in Microbial Diversity and Sustainable Utilization, Chiang Mai University Chiang Mai Thailand; 13 Center of Excellence in Microbial Diversity and Sustainable Utilization, Chiang Mai University, Chiang Mai 50200, Thailand Office of Research Administration, Chiang Mai University Chiang Mai Thailand; 14 Office of Research Administration, Chiang Mai University, Chiang Mai 50200, Thailand Faculty of Technology, Rajarata University Mihintale Sri Lanka; 15 Department of Health Sciences, Faculty of Medicine and Health Sciences, University of Mauritius, Reduit 80837, Mauritius Tropical Microbiology Research Foundation Pannipitiya Sri Lanka; 16 Department of Zoology, College of Science, King Saud University, Riyadh P.O. Box 2455, Saudi Arabia Patanjali Research Institute Uttarakhand India; 17 School of Pharmacy, Zunyi Medical University, Zunyi, Guizhou Province 563000, China Faculty of Medicine and Health Sciences, University of Mauritius Reduit Mauritius

**Keywords:** New family, *
Pleosporales
*, *
Rostriconidium
*, *
Sporidesmioides
*, taxonomy, *
Torulaceae
*, Western Ghats

## Abstract

This study reappraised the taxonomy of the genus *Rostriconidium*, and introduced a new species, *Rostriconidium synnematicum*, based on an integrated molecular and morphological taxonomic approach. This new taxon is the only synnematous species within *Rostriconidium* and forms a sister lineage to other *Rostriconidium* species in *Torulaceae*, as supported by phylogenetic analyses of ITS, LSU, SSU, *rpb*2 and *tef1*-*α* sequences using Maximum Likelihood and Bayesian Inference methods. Additionally, a new combination *Rostriconidium yunnanensis* is proposed under *Rostriconidium*. The study addresses the taxonomic complexities of four morphologically similar but phylogenetically distinct genera (*Neopodoconis*, *Pseudohelminthosporium*, *Rostriconidium*, and *Sporidesmioides*) within the *Pleosporales*. There are a few taxonomic challenges in resolving relationships among these genera, given their polyphyletic nature and overlapping morphologies. In this study, advanced mycological studies were conducted to resolve the generic circumscription of *Sporidesmioides*. In this study, we propose a new familial lineage, *Sporidesmioidesaceae*, to accommodate the monotypic species *S. thailandica*, based on phylogenetic analyses of a multi-locus dataset (ITS, LSU, SSU, *rpb*2, and *tef1*-*α*). The results also accommodate *Pseudohelminthosporium* within *Neomassarinaceae*, rather than accepting the synonymization of *Pseudohelminthosporium clematidis* under *Neopodoconis ampullacea*. The conspecific nature of *P. clematidis* and *N. ampullacea* warrants a future type-based study to validate them. These findings underscore the critical importance of examining type specimens (morphological and multi-locus sequence analysis) before proposing synonymy among cryptic species and genera. This study also revalidated the placement of the recently proposed family *Megacapitulaceae* and provided their multigene sequence information.

## Introduction

*Pleosporales* represents the largest order within *Dothideomycetes*, encompassing around 34% of dothideomycetous fungi (Hyde et al. 2013; Liu et al. 2017; Pem et al. 2019; Hongsanan et al. 2020; Thiyagaraja et al. 2025). Species within *Pleosporales* are cosmopolitan in distribution, occurring globally as epiphytes, saprobes, endophytes, parasites, pathogens, hyperparasites on fungi or insects, and even as lichenised fungi (Zhang et al. 2012; Hongsanan et al. 2020; Mapook et al. 2020). According to Hyde et al. (2024), the order includes 92 families and about 591 genera. Recently, Rajeshkumar et al. (2024) introduced *Megacapitulaceae* and Wanasinghe et al. (2025) introduced *Mangifericomitaceae*, thus raising the number to 94. *Torulaceae*, a family within *Pleosporales*, was initially described by Corda (1829), with *Torula* as the type genus. According to Hyde et al. (2024), the *Torulaceae* currently comprises seven genera: *Cylindrotorula*, *Dendryphion*, *Neotorula*, *Rostriconidium*, *Rutola*, *Sporidesmioides*, and *Torula*. The genus *Rostriconidium* was established by Su et al. (2018), with *Rostriconidium aquaticum* as the type species. The genus is characterised by cylindrical, monotretic or polytretic conidiogenous cells and dark conidia that form pyriform to rostrate with a thick basal scar and a pale cell above it. The genus *Sporidesmioides* was introduced by Li et al. (2016), with *Sporidesmioides thailandica* as the type species. Genus is characterised by dematiaceous conidiophores, polyblastic conidiogenous cells, and ampulliform, septate, and dark brown conidia with paler cells at ends. Based on a combined sequence data from LSU, SSU, *tef1*-α, and *rpb*2 loci, Li et al. (2016) initially placed *Sporidesmioides* within *Torulaceae*. However, with the inclusion of additional taxa from families such as *Roussoellaceae* and *Thyridariaceae* of *Pleosporales*, *Sporidesmioides* was later found to form a distinct clade, separate from *Torulaceae* (Wang et al. 2023).

In their preprint (not a validly published work and therefore invalid), Qiu et al. (2022) synonymised *Rostriconidium* and *Sporidesmioides* under the genus *Neopodoconis* based on morphological similarities. Their phylogenetic inferences were based solely on the combined LSU and SSU loci. They introduced five new species: *Neopodoconis jiangxiensis*, *N. meilingensis*, *N. obclavata*, *N. saprophyticus*, and *N. sinensis*. Phylogenetically, *N. meilingensis* and *N. saprophyticus* grouped with *Rostriconidium* species within *Torulaceae*, while *N. jiangxiensis*, *N. obclavata*, and *N. sinensis* formed a sister subclade to *Sporidesmioides*, basal to *Roussoellaceae*. They considered the genus *Neopodoconis* to be polyphyletic. Subsequently, Wang et al. (2023) accepted the taxonomic proposals of Qiu et al. (2022) despite their preprint status and introduced a new species, *Neopodoconis yunnanensis*. Their analyses revealed two distinct phylogenetic clades within *Neopodoconis*: Clade 1, comprising *N. aquatica* (≡ *R. aquaticum*), *N. cangshanense* (≡ *R. cangshanense*), *N. pandanicola* (≡ *R. pandanicola*), *N. saprophyticus*, and *N. yunnanensis*, formed a monophyletic group under *Torulaceae*. Clade 2, comprising *N. jiangxiensis*, *N. meilingensis*, *N. obclavata*, *N. sinensis*, and *N. thailandica* (≡ *S. thailandica*), was placed outside *Torulaceae*. Despite their morphological similarity, the clades are phylogenetically unrelated and occupy distinct familial placements.

More recently, Li et al. (2024) confirmed this separation: *N. meilingensis*, *N. sinensis*, *N. saprophyticus*, and *N. yunnanensis* clustered with *Rostriconidium* in *Torulaceae*, while *N. jiangxiensis* and *N. obclavata* grouped with *Sporidesmioides* as a separate lineage. Based on molecular delimitation and phylogenetic evidence, they tentatively resurrected the genera *Rostriconidium* and *Sporidesmioides* until the generic circumscription of *Neopodoconis* is resolved through type-based studies. Meanwhile, Koukol and Delgado (2021) synonymised *Pseudohelminthosporium clematidis* (Phukhamsakda et al. 2020) with *Neopodoconis ampullacea*, the type species of *Neopodoconis* originally described by Rifai (2008). Consequently, Koukol and Delgado (2021) placed *Neopodoconis* under *Neomassarinaceae*. But their placement was based solely on morphological comparisons of type material, without any phylogenetic support.

In this study, we have collected a new species of *Rostriconidium* from the Western Ghats region of India and formally reinstate *Rostriconidium* as a valid genus within the family *Torulaceae*. We also propose a new family to accommodate *Sporidesmioides* and resurrect *Pseudohelminthosporium clematidis* as that study was not supported by molecular evidence. This study also revalidated the placement of the recently proposed family *Megacapitulaceae* within *Pleosporales* and provided their multigene sequence information.

## Materials and methods

### Collection, isolation, and morphology

As part of an exploration of lignicolous microfungal diversity in the Western Ghats of India, surveys were conducted to assess the diversity of microfungi in decaying litter samples. Samples were collected during the post-monsoon season (August 5, 2023) from semi-evergreen natural forests in Tamhini Village, Mulshi Area (18.489522°N, 73.513039°E) of Maharashtra, India. Conidia were isolated directly from the unidentified decaying twig and observed using a binocular stereomicroscope (Olympus SZX16 with Digi-CAM, Japan). Single-conidial cultures were established on 2% malt extract agar plates (MEA; HiMedia, Mumbai, India) as described by Rajeshkumar et al. (2021, 2023). Microscopic observations were made with a binocular stereomicroscope (Olympus SZX16 with Digi-CAM, Japan) and a Carl Zeiss (AXIO Imager 2, Oberkochen, Germany) compound microscope equipped with Nikon Digital Sight DS-Fi1 and AxioCam MRc5 cameras driven by AxioVision Rel 4.8 software (AXIO Imager 2, Oberkochen, Germany). Conidia and conidiophores were mounted in lactic acid and cotton blue and measured using an ocular micrometer (confirmed with software available with the Zeiss microscope), with 30 observations per structure. For scanning electron microscopy (SEM), synnemata with conidia were fixed to aluminum stubs using double-sided carbon tape and sputter–coated with gold-palladium. SEM observations and photography were made with Carl Zeiss EVO 50 Scanning Electron Microscope (Carl Zeiss, Oberkochen, Germany) at Agharkar Research Institute, Pune. Culture characteristics were recorded after two weeks of incubation on MEA and potato dextrose agar (PDA) (HiMedia, Mumbai, India). Methods for inoculations, incubation conditions, and microscopic slide preparations followed those described by Senanayake et al. (2020). Colour codes used in descriptions refer to Kornerup and Wanscher (1978). Herbarium specimens were deposited in the Ajrekar Mycological Herbarium (**AMH**), and the culture is preserved at the National Fungal Culture Collection of India (**NFCCI**, WDCM-932) at the Agharkar Research Institute, Pune, India. Fungal nomenclatural and taxonomic details were submitted to Index Fungorum.

### DNA extraction, amplification, and phylogenetic analyses

Colonies were grown on MEA plates for two weeks, and genomic DNA was extracted according to the protocols described by Aljanabi and Martinez (1997). The 5' end of the 28S large subunit nrRNA (LSU) gene region was amplified using primer pairs LR0R and LR7 (Vilgalys and Hester 1990). The 5' end of the 18S small subunit nrRNA (SSU) gene was amplified using the primers NS1 and NS4 (White et al. 1990). Protein coding of the partial RNA polymerase II second largest subunit (*rpb*2) and the partial translation elongation factor 1-alpha gene (*tef1*α) were amplified using the primer pairs fRPB2-5f/fRPB2-7cR (Liu et al. 1999) and EF1-983F/EF1-2218R (Rehner and Buckley 2005), respectively. The amplification was performed in a 25 μL reaction volume containing 9.5 μL ddH_2_O, 12.5 μL 2× Taq PCR Master Mix with blue dye (Sangon Biotech, Shanghai, China), 1 μL of DNA template, and 1 μL of each primer (10 μM). The amplification conditions for LSU consisted of an initial denaturation at 94 °C for 3 min, followed by 35 cycles of denaturation at 94 °C for 40 s, annealing at 52 °C for 45 s, and extension at 72 °C for 60 s, with a final extension at 72 °C for 10 min, and a hold at 4 °C (White et al. 1990). The amplification conditions for SSU consisted of an initial denaturation at 94 °C for 3 min, followed by 35 cycles of denaturation at 94 °C for 40 s, annealing at 50 °C for 45 s, and extension at 72 °C for 60 s, with a final extension at 72 °C for 10 min, and a hold at 4 °C (White et al. 1990). The amplification conditions for *tef1*-*α* were carried out using a touchdown PCR protocol, consisting of an initial denaturation at 94 °C for 2 min, followed by 9 cycles of denaturation at 94 °C for 2 min, with annealing temperatures decreasing from 66 °C to 56 °C (66, 65, 64, 63, 62, 61, 60, 59, 58, 57 and 56 °C; 30 s each), and extension at 72 °C for 1 min, with a final extension at 72 °C (Rehner and Buckley 2005). The amplification conditions for *rpb2* were performed using a touch-up PCR protocol, consisting of an initial denaturation at 95 °C for 5 min, followed by 35 cycles of denaturation at 94 °C for 1 min, annealing at progressively increasing temperatures of 54 °C, 58 °C, and 60 °C (2 min each), and extension at 72 °C for 2 min, with a final extension at 72 °C for 10 min and a hold at 4 °C (Liu et al. 1999). After multiple attempts, amplification of the internal transcribed spacer (ITS) of the nuclear ribosomal DNA (ITS1, 5.8S, ITS2) was unsuccessful. The PCR products were purified using a StrataPrep PCR Purification Kit (Agilent Technologies, CA, USA) and sequenced with the same primers in the BigDye Terminator v. 3.1 Cycle Sequencing Kit (Applied Biosystems, MA, USA). Sequencing reactions were performed in both forward and reverse directions (bidirectional sequencing) and run on ABI PRISM 3100 Genetic Analyzer (Applied Biosystems, MA, USA).

The NCBI GenBank nucleotide sequence database was searched using MegaBLAST (Morgulis et al. 2008) to identify the most closely matching sequences. Two different datasets were analysed in this study. The first dataset (Dataset 1) evaluated familial relationships within the *Pleosporales* and was based on a larger taxon sampling, incorporating representative genera with DNA sequence data from families. Another dataset (Dataset 2) evaluated the phylogenetic relationships within *Torulaceae*, as we introduce a new species to the family. Dataset 1 was mainly compiled (Suppl. material 2) based on sequences published by Gomdola et al. (2024) and Rajeshkumar et al. (2024). Dataset 2 (Suppl. material 2) was compiled mainly based on sequences published by Wang et al. (2023) and Li et al. (2024). Both Dataset 1 and Dataset 2 were aligned with our newly obtained sequences using MAFFT v. 7.453 (Katoh et al. 2019) by selecting the G-INS-i option. The resulting alignments were manually inspected and edited in BioEdit v.7.0.9.0 (Hall 1999), and ambiguously aligned regions were excluded to minimize phylogenetic noise. Two phylogenetic trees are presented, one with representatives of families in *Pleosporales* and the other with all *Torulaceae* members.

Members of *Tubeufiales* were chosen as the outgroup for ordinal-level phylogenetic analyses based on a combined LSU-ITS-SSU-*tef1*α-*rpb*2 sequence dataset from *Pleosporales*. *Occultibambusa bambusae* MFLUCC 13-0855 and *Neooccultibambusa thailandensis* MFLUCC 16-0274 were chosen as an outgroup for the combined LSU-ITS-SSU-*tef1α*-*rpb2* analyses of *Torulaceae* members. Alignments were concatenated using SequenceMatrix v.1.9 (Vaidya et al. 2011). AliView (Larsson 2014) was used to convert the alignment file to PHYLIP and NEXUS formats for Maximum Likelihood (ML) and Bayesian analyses, respectively. Phylogenetic analyses of concatenated datasets (LSU-ITS-SSU-*tef1α*-*rpb2*) were performed using the Maximum Likelihood (ML) method and Bayesian posterior probability (BY-PP). ML analyses were performed using IQ-TREE v.3.0.1 (Nguyen et al. 2015; Kalyaanamoorthy et al. 2017; Minh et al. 2020) for concatenated datasets (LSU-ITS-SSU-*tef1α*-*rpb2*). IQ-TREE was used to determine the best-fit substitution model for each data set using the -m TESTNEW parameter, with model selection performed by ModelFinder (Kalyaanamoorthy et al. 2017) based on the Bayesian Information Criterion (BIC), and the selected models representing those with the lowest BIC scores among the candidate models tested. Support at the nodes was calculated from 1,000 non-parametric bootstrap replicates. GAMMA Model parameters were estimated to an accuracy of 0.1000000000 log-likelihood units. Every 100^th^ tree was saved. The Bayesian posterior probability (BY-PP) analysis was performed using MrBayes v. 3.2.7a (Ronquist et al. 2012), using the parameter settings of two parallel runs of four chains each, run for 100 million generations but with the stop value set at 0.01, the temperature set at 0.2, and the sample frequency every 10^th^ generation. The 50% majority-rule consensus tree was created from the remaining trees after the first 25% of sampled trees were excluded as burn-in. The resulting trees were illustrated with Figtree v1.3.1 (Rambaut 2010), and MEGA 7 (Kumar et al. 2016), and the tree layout was created in Microsoft PowerPoint. The newly generated DNA sequences from this study have been deposited in GenBank. Species recognition was done based on the guidelines provided by Chethana et al. (2021) and Pem et al. (2021). Gene and site concordance factor analyses were conducted using IQ-TREE v3.0.1 to evaluate phylogenetic congruence among loci. The Maximum Likelihood (ML) tree inferred from the concatenated dataset was used as the reference tree. Gene concordance factors (gCF) were calculated based on a set of individual gene trees, while site concordance factors (sCF) were estimated using 1,000 randomly sampled quartets per branch (Minh et al. 2020).

## Results

### Phylogeny

**Figure 1.**
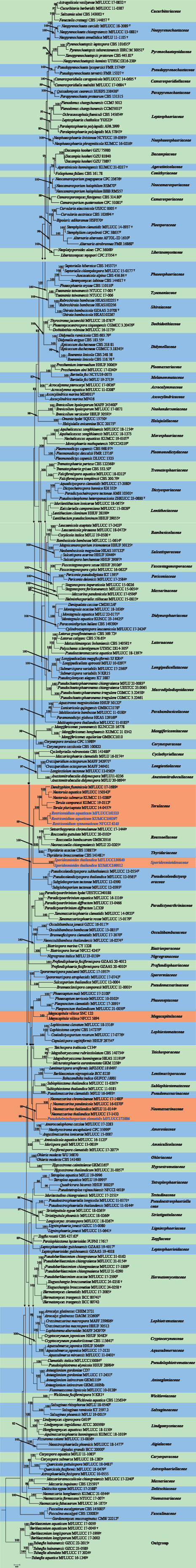
Maximum likelihood (ML) phylogenetic tree obtained from an IQ-TREE analysis of representative species in 93 families of *Pleosporales* based on LSU, ITS, SSU, *tef*-α, and *rpb*2 sequence data. Strains under study are shown in blue bold text and their respective families are in orange blocks. Families are indicated on the right side of the tree. Branch support values from 1000 non-parametric bootstraps for IQ-TREE (ML-BS) are shown at the nodes (ML-BS≥80%). The tree is rooted with members of *Tubeufiales*.

**Figure 2.**
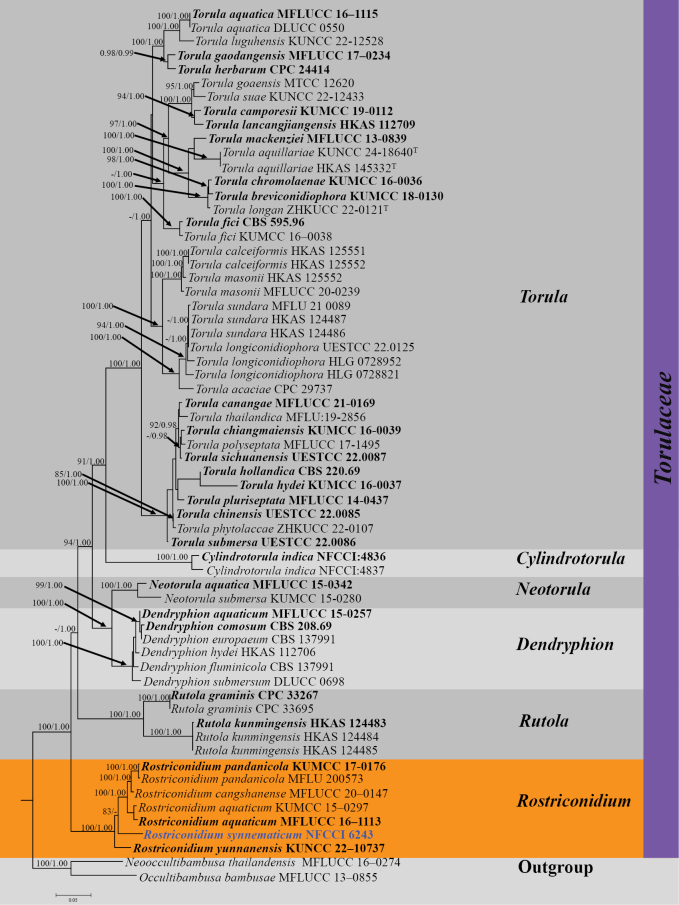
Maximum Likelihood (ML) phylogenetic tree obtained from an IQ-TREE analysis of species from *Torulaceae* based on LSU, ITS, SSU, *tef1α*, and *rpb*2 sequence data. Strain of the species under study is shown in blue bold text, and the type strains are in bold black. Family is indicated on the right side of the tree in blocks. Branch support values from 1000 non-parametric bootstraps for IQ-TREE (ML-BS) and posterior probability values from the Bayesian analysis (BY-PP) are shown at the nodes (ML-BS≥70%/BY-PP≥0.9). The tree is rooted with *Occultibambusa bambusae* MFLUCC 13-0855 and *Neooccultibambusa thailandensis* MFLUCC 16-0274.

Based on a MegaBLAST search of our new strain in NCBI’s GenBank nucleotide database, the closest matches using LSU, SSU, *tef1*-α, and *rpb*2 are given in Table 1. The final combined LSU-ITS-SSU-*tef1*α-*rpb*2 dataset 1 (order-level analyses) comprised 4,382 characters, including all alignment gaps for 300 strains and the outgroups. The obtained ML tree (Fig. 1) had a log-likelihood of -154487.525. The matrix contained 3,386 distinct alignment patterns, 2,171 parsimony-informative, 653 singleton sites, and 1,558 constant sites. Parameters for the GTR+F+R8 model of the combined LSU-ITS-SSU-*tef1α*-*rpb2* dataset were as follows: estimated base frequencies A = 0.245, C = 0.248, G = 0.272, T = 0.236; substitution rates AC = 1.48807, AG = 3.87262, AT = 1.66629, CG = 1.16329, CT = 7.83329, GT = 1.00000; gamma distribution shape parameter α = 0.594. The final combined LSU-ITS-SSU-*tef1*-α-*rpb*2 dataset 2 (family-level analyses) comprised 5,148 characters, including all alignment gaps for 63 strains and the outgroups. The obtained ML tree (Fig. 2) had a log-likelihood of -27071.853. The matrix contained 1,667 distinct alignment patterns, 1,020 parsimony-informative, 291 singleton sites, and 3,837 constant sites. Parameters for the TIM2+F+I+G4 model of the combined LSU-ITS-SSU-*tef1α*-*rpb2* dataset were as follows: estimated base frequencies A = 0.246, C = 0.260, G = 0.271, T = 0.223; substitution rates AC = 1.49444, AG = 3.06877, AT = 1.49444, CG = 1.00000, CT = 6.80060, GT = 1.00000; gamma distribution shape parameter α = 0.552. The final ML concatenated tree with superimposed BY-PP values is shown in Figs 1, 2.

**Table 1.** BLAST result of LSU, SSU, *tef1*-α, and *rpb*2 sequences of *Rostriconidium synnematicum* (AMH 10845).

Locus	Organism Name	Strain/Isolate	GenBank accession	Identities	Gaps
**LSU**	*Torula goaensis*	MTCC 12620	NG_060016	1277/1322(97%)	7/1322(0%)
	*Torula herbarum*	MUT<ITA>:3038	MG980414	1279/1326(96%)	6/1326(0%)
	*Dendryphion hydei*	KUN-HKAS:112706	MW879527	1268/1310(97%)	7/1310(0%)
**SSU**	*Rostriconidium aquaticum*	GZCC19-0424	MW134601	957/965(99%)	0/965(0%)
	*Rostriconidium pandanicola*	MFLU:20-0573	MW010282	947/954(99%)	0/954(0%)
	*Dendryphion* sp.	DL-C40a	PQ607977	966/985(98%)	0/985(0%)
***tef1*-α**	*Rostriconidium aquaticum*	KUMCC 15-0297	MG207995	858/889(97%)	0/889(0%)
	*Rostriconidium aquaticum*	GZCC:19-0424	OP473011	868/908(96%)	0/908(0%)
	*Rostriconidium yunnanensis*	KUNCC22-10737	OP471613	844/885(95%)	1/885(0%)
***rpb*2**	*Neopodoconis yunnanensis*	KUNCC 22-10737	OP476726	764/830(92%)	0/830(0%)
	*Rostriconidium aquaticum*	UESTCC 01.0192	PV170690	791/862(92%)	1/862(0%)
	*Rostriconidium cangshanense*	MFLU:20-0752	MW012636	895/975(92%)	1/975(0%)

In our phylogenetic analyses of all families in *Pleosporales* (Fig. 1), representative taxa from 93 families, including our newly introduced family were included. *Diademaceae* and *Lizoniaceae*, which also belong to *Pleosporales* but lack molecular data, were omitted from analyses. The placement of *Sporidesmioides thailandica* has remained a source of confusion since its introduction into *Torulaceae* as a basal lineage to *Roussoellaceae* by Li et al. (2016). Later, Su et al. (2018) and Tibpromma et al. (2018) also placed *Sporidesmioides* under *Torulaceae* while introducing *Rostriconidium* into the family; in both analyses, *Sporidesmioides* was placed as a separate basal lineage to all other members of *Torulaceae*. Recently, He et al. (2024) also placed *Sporidesmioides* under *Torulaceae*, as a paradox lineage. Wang et al. (2023) and Li et al. (2024) placed *Sporidesmioides*, basal to *Roussoellaceae*, *Thyridariaceae*, and *Torulaceae* as a separate clade. In our familial level phylogenetic analyses of all Pleosporales members, *Sporidesmioides* is placed as a separate lineage basal to *Roussoellaceae*, *Thyridariaceae*, and *Torulaceae*, substantiating the placement of Wang et al. (2023) and Li et al. (2024) with significant statistical support (ML-BS = 99%; Fig. 1). Here, we treat *Sporidesmioides* under a new family, *Sporidesmioidesaceae*, to clarify its placement within *Pleosporales* since its original establishment. The resulting phylogenetic trees annotated with gene concordance factor (gCF) and site concordance factor (sCF) values are provided as Suppl. material 1.

Phylogenetic analyses of the LSU-ITS-SSU-*tef1*-α-*rpb*2 concatenated dataset 2 and individual datasets (ITS, LSU, *rpb*2, SSU, and *tef1*-α) supported the placement of the new species, *Rostriconidium synnematicum*, as a sister lineage to other *Rostriconidium* species with strong statistical support (ML-BS = 100%/BY-PP = 1.00) (Fig. 2). In our analyses, *R. aquaticum* MFLUCC 16-1113, *R. aquaticum* KUMCC 15-0297, *R. cangshanense* MFLUCC 20-0147, *R. pandanicola* KUMCC 17–0176, *R. yunnanensis* KUNCC 22-10737, and *R. synnematicum*AMH 10845 formed a separate clade under *Torulaceae* with strong statistical support (ML-BS = 100%/BY-PP = 1.00). We have not included *Neopodoconis jiangxiensis* (HJAUP C0947), *N. meilingensis* (HJAUP C0905), *N. obclavata* (HJAUP C0829), *N. sinensis* (HJAUP C0909), and *N. saprophyticus* (HJAUP C0830) in our phylogenetic analyses as these were not validly published in accordance with the International Code of Nomenclature (ICNafp).

This study reinstates the genus *Pseudohelminthosporium* introduced by Phukhamsakda et al. (2020). The taxonomic treatment of Koukol and Delgado (2021) to synonymise *Pseudohelminthosporium clematidis* MFLUCC 17-2086 under *Neopodoconis ampullacea* cannot be followed, as they synonymised the genus without any molecular studies. To state *Pseudohelminthosporium* congeneric to *Neopodoconis*, DNA data must be obtained from the type specimen of both genera, and phylogenetic analyses must validate it. This synonymisation should not be considered valid, as the type strain of *Neopodoconis ampullacea* is unavailable and thus the congeneric status could not be clarified. Our study itself showed that, although *Sporidesmioides* and *Rostriconidium* are morphologically similar, their molecular data distinguish them as two separate genera within different families. In our order-level phylogenetic analyses of all *Pleosporales* members, *Pseudohelminthosporium clematidis* MFLUCC 17-2086 formed a separate lineage basal to *Neomassarinaceae* with significant statistical support (ML-BS = 99%/BY-PP = 1.00). This is because we are treating *Neopodoconis* under a genus within *Neomassarinaceae*.

### Taxonomy

#### Sporidesmioidesaceae

Taxon classificationFungiPleosporalesSporidesmioidesaceae

Rajeshk., O.P. Sruthi, Jeewon, Karun., J.F. Li & Wijayaw. fam. nov.

5BBECA31-D034-563E-8CF9-21C2C5D7F359

Index Fungorum: IF904794

Fig. 3

##### Etymology.

Refers to the name of the type genus, *Sporidesmioides*.

**Figure 3.**
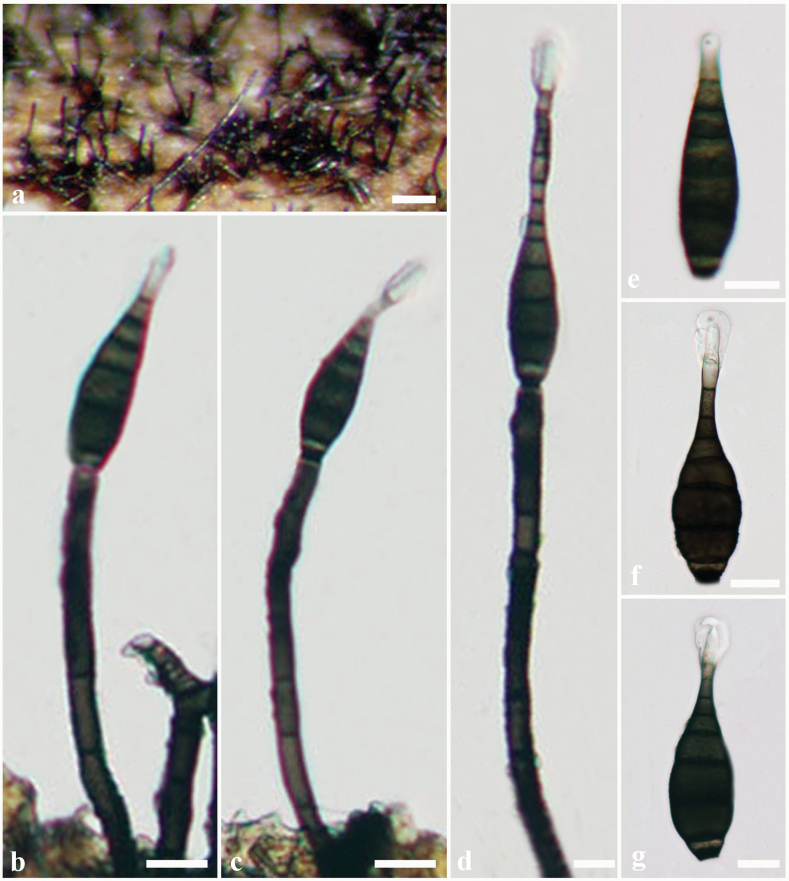
*Sporidesmioides thailandica* (MFLU 14-0827). **a** Pseudoparenchymatous stromata on stem of a dead herb. **b–c** Conidiophores with conidia on a pseudoparenchymatous stromata. **d** Conidiophores bearing conidia. **e–g** Conidia. Scale bars: **a** = 100 μm, **b–c** = 100 μm, **e–g** = 20 μm, **d** = 5 μm.

##### Classification.

*Pleosporales*, *Dothideomycetes*, *Ascomycota*.

##### Description.

***Saprobic*** on the plant litter of wood or herbs. **Sexual morph**: Undetermined. **Asexual morph**: Hyphomycetous. ***Conidiophores*** macronematous, mononematous, straight or curved, septate, smooth, brown to dark brown, unbranched, erect, single or caespitose, smooth, thick-walled, cylindrical, arising from a stromatic base akin to pseudoparenchymatous stromata. ***Conidiogenous cells*** polyblastic, integrated, determinate or percurrent, terminal, sometimes becoming intercalary sympodial, often dark and prominent, cylindrical or doliiform. ***Conidia*** acrogenous, solitary, dry, ampulliform to obclavate, rostrate, straight or curved, conicotruncate at the base, septate, brown to dark brown, with paler cells at the end, smooth or verruculose to echinulate, subhyaline at the apex, thick-walled, with or without hyaline sheath at the apex. Conidial secession schizolytic (adopted from Li et al. 2016).

##### Type genus.

*Sporidesmioides* J.F. Li, Phookamsak & K.D. Hyde.

##### Host and distribution.

on stem of a dead herb in Thailand (Chiang Rai Province).

##### Notes.

*Sporidesmioidesaceae*, our newly established monotypic family, comprises the single genus *Sporidesmioides* with the type species *S. thailandica*. The family is known only from its asexual morph (Li et al. 2016). Morphologically, *S. thailandica* shows superficial resemblance to species of *Neopodoconis* and *Rostriconidium* in conidial shape and pigmentation; however, *Sporidesmioidesaceae* differs fundamentally in its mode of conidiogenesis, which is strictly polyblastic, whereas *Neopodoconis* and *Rostriconidium* exhibit mono or polytretic conidiogenesis. In phylogenetic analyses based on combined LSU, ITS, SSU, *tef1*-*α*, and *rpb2* gene datasets, *S. thailandica* forms a distinct clade sister to *Roussoellaceae*, *Thyridariaceae*, and *Torulaceae*, supporting its recognition as a separate family within the order *Pleosporales*, class *Dothideomycetes*.

When compared with *Torulaceae*, *Sporidesmioidesaceae* differs in several key morphological characters. Both families are known only from asexual morphs; however, conidiophores in *Torulaceae* are erect, subcylindrical, branched or unbranched, and arise from an immersed mycelium, whereas *Sporidesmioidesaceae* produces erect, cylindrical, and unbranched conidiophores arising from a stromatic base. Conidiogenous cells in *Torulaceae* are mono or polyblastic, doliiform to ellipsoid or clavate, smooth to verruculose, sometimes cupulate, while those of *Sporidesmioidesaceae* are strictly polyblastic, integrated, and frequently pigmented. In addition, conidia in *Torulaceae* are mostly subcylindrical, phragmosporous, brown, septate, and commonly arranged in branched chains, contrasting with the solitary, ampulliform to obclavate conidia of *Sporidesmioidesaceae*.

*Sporidesmioidesaceae* also differs markedly from *Roussoellaceae* by well-developed sexual morphs and coelomycetous asexual morphs, whereas *Sporidesmioidesaceae* is strictly hyphomycetous and lacks a known sexual morph. The asexual morphs of *Roussoellaceae* are highly variable, with pycnidial, stromatic conidiomata that may be cytoplea-like, melanconiopsis-like, neomelanconium-like, or cyclothyrium-like. In contrast, Sporidesmioidesaceae has mononematous conidiophores. Furthermore, conidiogenous cells in *Roussoellaceae* are holoblastic, annellidic, discrete, and often hyaline, whereas those in *Sporidesmioidesaceae* are integrated, polyblastic. Conidia in *Roussoellaceae* are globose to ellipsoidal and range from hyaline to dark brown, contrasting with the consistently pigmented, pyriform conidia of *Sporidesmioidesaceae*.

In comparison with *Thyridariaceae*, *Sporidesmioidesaceae* is distinguished by the absence of sexual morphs and by its hyphomycetous asexual morph. *Thyridariaceae* typically possesses prominent sexual morphs and cyclothyrium-like asexual morphs with pycnidial conidiomata. Conidiogenous cells in *Thyridariaceae* are holoblastic or polyblastic, and conidia are generally brown, smooth-walled, and septate, but they are produced within conidiomata rather than directly on conidiophores as in *Sporidesmioidesaceae*. These diagnostic morphological characters, together with strong multi-gene phylogenetic support, justify the introduction of *Sporidesmioidesaceae* as a new family within *Pleosporales*.

### *Torulaceae* Corda (Corda 1829)

#### Rostriconidium synnematicum

Taxon classificationFungiPleosporalesTorulaceae

Rajeshk., O.P. Sruthi, Karun., R. K. Verma & Wijayaw. sp. nov.

76BBFF27-9AC7-52E6-8EE1-CC9680286721

Index Fungorum: IF904795

Figs 4, 5

##### Etymology.

Refers to the synnematous nature of fungus.

**Figure 4.**
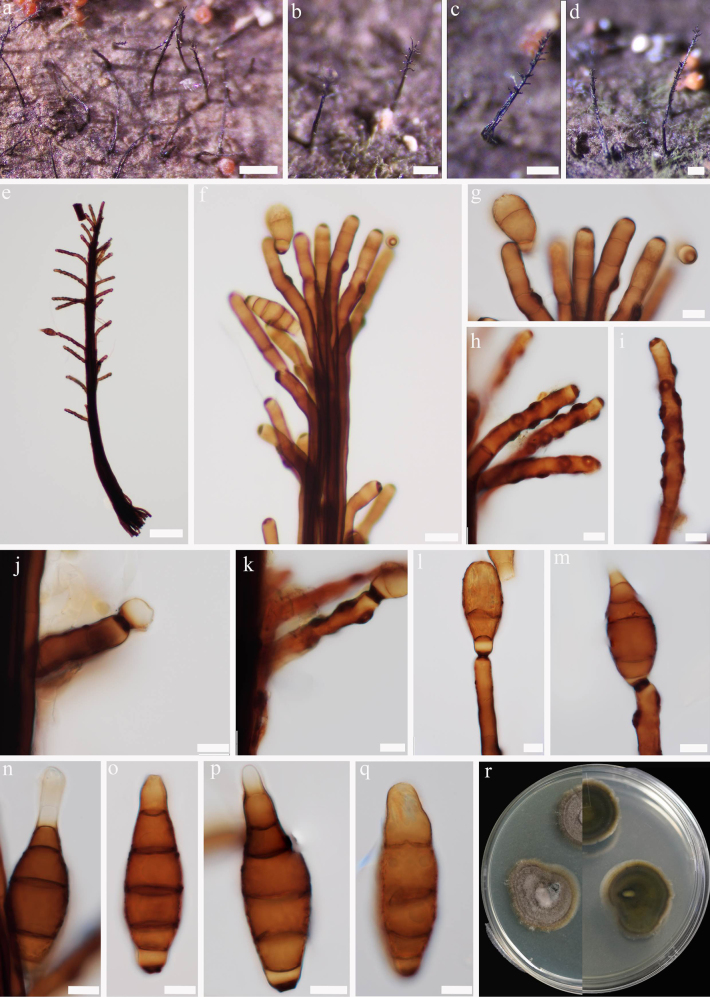
*Rostriconidium synnematicum* (AMH 10845). **a–d**. Synnemata on natural substrate; **e**. Synnemata with attached conidia; **f, g**. Conidia and terminal conidiogenous cells; **h, i**. Polytretic conidiogenous cells showing pores; **j, k**. Young developing conidia attached to the conidiogenous cells; **l, m**. Developing conidia attached with terminal conidiogenous cells; **n–q**. Mature conidia with variable shape; **r**. Colonies on MEA obverse and reverse. Scale bars: 500 μm (**a**); 200 μm (**b–d**); 100 μm (**e**); 20 μm (**f**); 10 μm (**g–q**).

**Figure 5.**
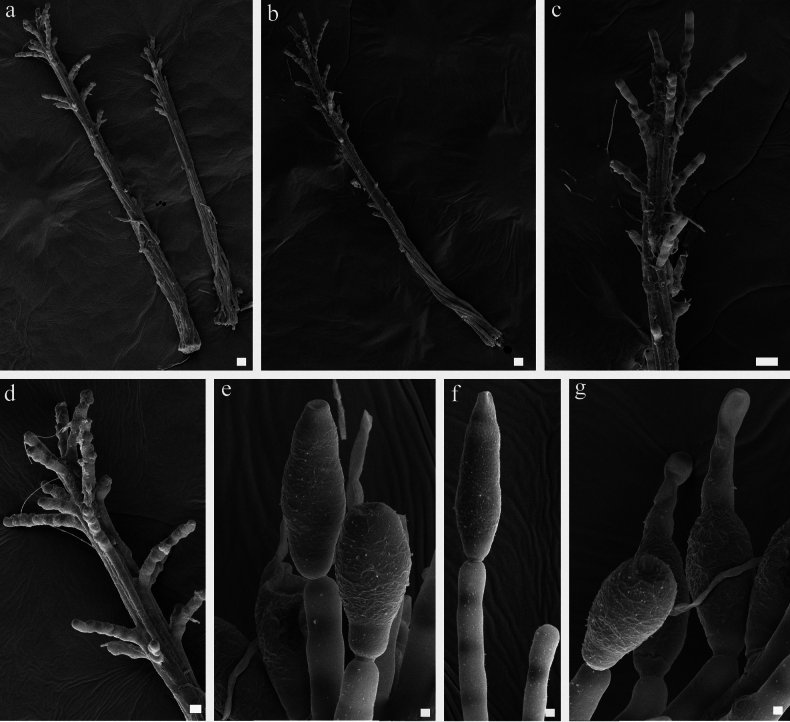
*Rostriconidium synnematicum* (AMH 10845) on SEM. **a–d**. Synnemata; **e–g**. Conidia attached with terminal conidiogenous cells. Scale bars: 20 μm (**a–c**); 10 μm (**d**); 2 μm (**e–g**).

##### Holotype.

AMH 10845.

##### Description.

***Saprobic*** on submerged decaying wood. **Sexual morph**: Undetermined. **Asexual morph: *Colonies*** grow on the surface of the substrate, black, with indeterminate synnemata. ***Mycelium*** immersed in the substrate, composed of pale brown, septate, unbranched hyphae. ***Synnemata*** 720–1450 × 8.2–17.5 μm (*x–* = 1150 × 12 μm, n = 30) dark brown to black, smooth-walled, septate, unbranched, straight or slightly flexuous. ***Conidiogenous cells*** enteroblastic, monotretic or polytretic, sympodial, integrated, pore dimension 4–6.5 × 4–7.5 μm. ***Conidial secession*** schizolytic. ***Conidia*** 44–70 × 13–22 μm (*x–* = 56 × 18 μm, n = 30), solitary, smooth, dry, acropleurogenous, young conidia obpyriform and fusiform at maturity, dark brown, pale or light brown at the apex, rostrate, guttulate, 4–6-septate, dark bands at the septa, black truncate scar at base.

##### Culture characteristics.

Colonies on MEA at 25 ± 2 °C after 20 d slow growing, reaching 28–30 mm diam., initially white (1A1), becoming greyish brown (5D3) to brownish grey (5D2) with age, reverse olive brown (4D4), to brownish grey (5D2). Colonies circular, flat, velvety, with irregularly lobate margins that are umbonate at the centre.

##### Material examined.

India. • Maharashtra, Tamhini Ghats, Mulshi Village, 18.489522°N, 73.513039°E, on unidentified decaying wood, 05 August 2023, OP Sruthi & KC Rajeshkumar, **holotype**—AMH 10845, *ex*-*type* living culture NFCCI 6243.

##### Notes.

Our new collection (AMH 10845) differs from all other accepted species of *Rostriconidium* by its larger synnemata (720–1450 × 8.2–17.5 μm). Conidia of *R. synnematicum* (44–70 × 13–22 μm) are smaller than the conidia of other species of *Rostriconidium*, and they also differ in the number of conidial septa. Conidiogenesis in *R. synnematicum* is monotretic or polytretic, as reported in the type species *R. aquaticum*. In the remaining *Rostriconidium* species, conidiogenesis is described as mono- or polyblastic, instead giving importance to the trectic conidiogenesis. Phylogenetically, the new collection represents a new lineage within *Torulaceae*, sister to the clade comprising *Rostriconidium pandanicola*, *R. cangshanense*, and *R. aquaticum*, and is supported by statistical evidence (ML-BS = 100%, BY-PP = 1.00) (Figs 1, 2). Hence, we introduce our new collection as a new species of *Rostriconidium* s.str. i.e., *R. synnematicum*. A detailed synopsis table of all phylogenetically accepted species under *Rostriconidium* is given in Table 2.

**Table 2.** A comparison table of the species reported under *Rostriconidium*, and *Sporidesmioides*.

Genus	Species	Specimen voucher	Conidiophores characteristics & dimensions	Conidiogenous Cells characteristics & dimensions	Conidia characteristics & dimensions	References
1	*Rostriconidium aquaticum* Z.L. Luo, K.D. Hyde & Hong Y. Su	MFLU 17–1415	370–590 × 13–17 μm, macronematous, mononematous, single, erect, septate, smooth, straight or slightly flexuous, dark brown to black, pale pigment at apex.	Monotretic or polytretic, sympodial, integrated, terminal, cylindrical, dark brown.	134–180 × 22–26 μm, solitary, dry, fusiform to pyriform, rostrate at maturity, dark brown to black, with guttulate cells, thickly cicatrized at narrow, with a thick, black truncate scar at base and pale pigment cell above the scar, wide in the middle, narrowly cylindrical, obtuse, and subhyaline at apex, 8–9-septate, slightly constricted at some septa, smooth.	Su et al. (2018)
2	*Rostriconidium cangshanense* H.W. Shen, Z.L. Luo & Hong Y. Su	MFLU: 20-0572.	279–528 × 12–14 μm, macronematous, mononematous, single, septate, dark brown to black, smooth or rough-walled, unbranched, straight or slightly flexuous, light-color at the apex.	Polytretic, integrated, terminal, cylindrical, smooth, dark brown, with black circle-peak shape conidiogenous scars.	94–109 × 21–24 μm, dry, solitary, pyriform, fusiform to obclavate, rostrate, guttulate, dark brown to black, 6–8-septate, slightly constricted at some septa, with black, truncate, dark structure at the base with a light-colored cell above it, straight or slightly flexuous, smooth-walled, sometimes with hyaline sheath at the apex.	Shen et al. (2021) (Conidiogenesis is amended)
3	*Rostriconidium pandanicola* Tibpromma & K.D. Hyde	HKAS 99620	360–485 × 11–13 μm, macronematous, mononematous, scattered, dark brown to black, rough-walled, septate, unbranched, straight or slightly flexuous.	15–30 × 8.5–12 μm, polytretic, sympodial, integrated, terminal, later becoming intercalary, cylindrical, dark brown, smooth, with black conidiogenous scars.	55–110 × 18–26 μm, solitary, pale brown to dark brown, rostrate, 4–7-septate, with dark bands at the septa, slightly constricted at septa, with truncate, thick scar at base, narrower at base, flattened and paler towards the apex, short apex when young, long rostrate apex when mature, with hyaline thin sheath at the apex, granulate, smooth-walled.	Tibpromma et al. (2018); (Conidiogenesis is amended)
4	*Rostriconidium yunnanensis* (W.P. Wang, H.W. Shen & Z.L. Luo) Rajeshk., O.P. Sruthi, Karun., Jeewon, Wijayaw.	KUN-HKAS 121702	174–648 × 8.2–17.5 μm, macronematous, mononematous, concentrated, erect, dark brown to black, smooth-walled, septate, unbranched, straight or slightly flexuous, pale pigment at apex.	18–34 × 9–14 μm, monotretic or polytretic, integrated, terminal, cylindrical, smooth, dark brown to black.	100–155 × 23–38 μm, solitary, smooth, dry, pyriform to fusiform, dark brown to black, light brown at the apex, granular inclusions, rostrate, guttulate, 5–7-septate, dark bands at the septa, slightly cicatrized at narrow, black truncate scar at base and pale pigment cell above the scar, wide in the middle.	Wang et al. (2023); (Conidiogenesis is amended)
5	*Rostriconidium synnematicum* Rajeshk., O.P. Sruthi, Karun., R. K. Verma & Wijayaw.	AMH 10845	720–1450 × 8.2–17.5 μm, synnemata dark brown to black, smooth-walled, septate, unbranched, straight or slightly flexuous.	4–6.5 × 4–7.5 μm, monotretic or polytretic, sympodial, integrated, pore dimension.	44–70 × 13–22 μm, solitary, smooth, dry, young conidia obpyriform and fusiform at mature, dark brown, pale or light brown at the apex, rostrate, guttulate, 4–6 septate, dark bands at the septa, black, truncate scar at base.	This study
6	*Sporidesmioides thailandica* Jun F. Li, Phookamsak & K.D. Hyde	MFLU 14-0827	120–200 × 7–9 μm, macronematous, mononematous, scattered, dark brown to black, slightly paler at the apex, smooth, thick-walled, septate, unbranched, straight to curved, sometimes percurrently proliferating, arising from a stromatic base.	14.2–25.5 × 6.6–8 μm, polyblastic, integrated, terminal, later becoming intercalary, cylindrical, dark brown, smooth, with pustulate, black, conidiogenous scars on conidial secession.	62.5–80 × 20–25 μm, solitary, simple, dark brown to black, ampulliform, minutely verruculose, 6–7-septate, slightly constricted at some septa, with a thick, black, truncate scar at the base, narrower, flattened and paler towards the tip, with a flap-like, hyaline, thick sheath at the tip, with densely granulate cells.	Li et al. (2016)

### New combinations in *Rostriconidium* s. str.

#### Rostriconidium yunnanensis

Taxon classificationFungiPleosporalesTorulaceae

(W.P. Wang, H.W. Shen & Z.L. Luo) Rajeshk., O.P. Sruthi, Karun., Jeewon & Wijayaw. comb. nov.

548E6BD2-B8B7-5983-9936-7BA6EF0D4AFC

Index Fungorum: IF904796

Synonym: Neopodoconis yunnanensis W.P. Wang, H.W. Shen & Z.L. Luo, in Wang, Shen, Bao, Lu, Yang, Su & Luo, MycoKeys 99: 8 (2023).

##### Holotype.

KUN-HKAS 121702.

##### Description.

Original description, see Wang et al. (2023).

##### Notes.

Wang et al. (2023) introduced *Rostriconidium yunnanensis* as *Neopodoconis yunnanensis* by accepting the treatment of Qiu et al. (2022); a preprint paper has not been published validly. Here, we synonymise it under *Rostriconidium* based on multi-gene phylogenetic analyses (Fig. 2). *Rostriconidium yunnanensis* is morphologically distinct from *R. aquaticum* in having shorter but broader conidia (100–155 × 23–38 vs. 134–180 × 22–26 μm). Additionally, it has smooth conidiophores, lacks constriction at the septum, and has larger conidia compared to *R. pandanicola* (100–155 × 23–38 μm vs. 55–110 × 18–26 μm), different from *R. cangshanense* in terms of larger size (100–155 × 23–38 vs. 94–109 × 11–24 μm), and lacks a hyaline sheath in the apex of conidia. Based on morphology and multigene phylogeny, Wang et al. (2023) described it as a new species. In this study, based on a combined LSU, ITS, SSU, *tef1*-*α*, and *rpb2* dataset, *R. yunnanensis* KUNCC 22-10737 was placed as a separate lineage basal to all other *Rostriconidium* species with statistical support.

### *Neomassarinaceae* Mapook & K.D. Hyde

*Neomassarinaceae* was established by Mapook et al. (2020). Currently, the family comprises three genera, viz., *Neomassarina*, *Pseudohelminthosporium*, and *Szafranskia*. Family was established to accommodate the members of *Neomassarina*, and it is characterised by crest-like ostiolar necks, with a carbonaceous texture, cylindrical to cylindric-clavate asci and broad fusiform ascospores surrounded by a mucilaginous sheath (Thambugala et al. 2015; Mapook et al. 2020). The first asexual genus of the family *Pseudohelminthosporium* was introduced by Phukhamsakda et al. (2020) based on phylogenetic analyses of LSU, SSU, ITS, *tef1*-*α*, and *rpb2* datasets, and the genus is characterised by macronematous conidiophores, polytretic conidiogenous cells, and phragmosporous, euseptate conidia. Later, Crous et al. (2024) introduced another asexual genus, *Szafranskia*, which is characterised by pycnidial conidiomata with conidiophores reduced to conidiogenous cells and hyaline, subhyaline, or pale brown, aseptate, ellipsoid conidia. Phylogenetic analyses based on a combined ITS, LSU, *rpb2*, and *tef1*-*α* dataset placed it as a sister to *Pseudohelminthosporium* (Crous et al. 2024). Crous et al. (2024) accepted the treatment of Koukol and Delgado (2021), considering *Pseudohelminthosporium* as a synonym of *Neopodoconis*.

#### Pseudohelminthosporium

Taxon classificationFungiPleosporalesNeomassarinaceae

Phukhams. & K.D. Hyde, Index of Australian Fungi 53: 13 (2025)

6B9A457D-0CED-5AC1-9254-F124FB3170AB

= Pseudohelminthosporium Phukhams. & K.D. Hyde, in Phukhamsakda et al., Fungal Diversity 102: 59 (2020) (Nom. inval., Art. 40.1 (Shenzhen)).

##### Description.

***Saprobic*** on decaying wood or herbaceous plant material in terrestrial habitats. **Sexual morph**: Undetermined. **Asexual morph: *Colonies*** on host substrates, effuse, black, hairy, scattered, dark brown. ***Mycelium*** immersed in the substrate forming dark brown stroma-like aggregations. ***Conidiophores*** macronematous, simple, solitary, branched at the apex, stripes straight or flexuous, cylindrical, dark brown to reddish brown, multi-septate, with well-defined small pores at the apex, smooth or verruculose**. *Conidiogenous cells*** monotretic or polytretic, integrated, terminal on conidiophores, doliiform to oblong, pale brown. ***Conidia*** phragmosporous, acrogenous, broad fusiform or obclavate, dark brown to reddish brown, distoseptate when young, becoming euseptate at maturity, verrucose, dark brown bud scars, disjunctions present at the basal position, hyaline, elongate cells at the upper end of conidia, with guttules in each cell.

#### Pseudohelminthosporium clematidis

Taxon classificationFungiPleosporalesNeomassarinaceae

Phukhams. & K.D. Hyde, in Tan, Bishop-Hurley, Marney & Shivas, Index of Australian Fungi 53: 13 (2025)

86D9CDD7-2251-5EC8-AC49-D3753652F08A

Index Fungorum: IF07284

Facesoffungi Number: FoF07284

Fig. 6

= Pseudohelminthosporium clematidis Phukhams. & K.D. Hyde, in Phukhamsakda et al., Fungal Diversity 102: 59 (2020).
= Pseudohelminthosporium clematidis Phukhams. & K.D. Hyde, in Tan & Dearnaley, Index of Australian Fungi 52: 5 (2025).

##### Holotype.

MFLU 17–1494.

**Figure 6.**
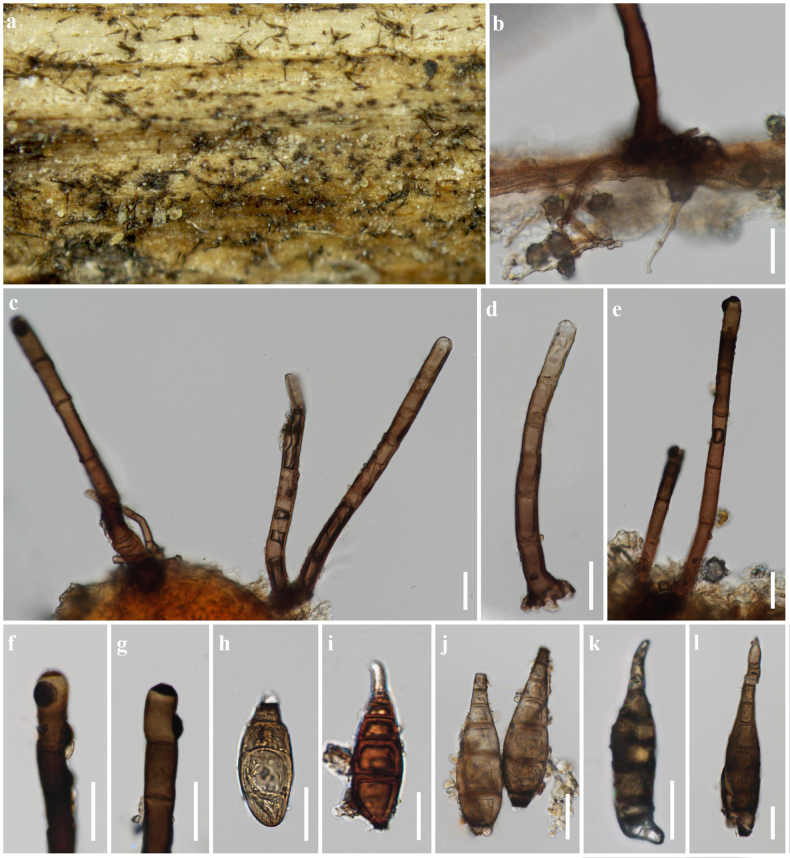
*Pseudohelminthosporium clematidis* (MFLU 17–1494, holotype). **a**. Conidiophores on natural substrate; **b**. Mycelia immersed in the host and the base of the conidiophore; **c–e**. Mononematous conidiophores; **f, g**. Conidiogenous cells with small pores at the apex; **h–l**. Conidia. Scale bars: 20 μm (**b–l**).

**Description. *Saprobic*** on decaying wood or herbaceous plant material in terrestrial habitats. **Sexual morph**: Undetermined. **Asexual morph: *Colonies*** on *Clematis sikkimensis* effuse, black, hairy, scattered, dark brown. ***Mycelium*** immersed, on the substrate surface forming stroma-like aggregations of dark brown sheet. ***Conidiophores*** 125–435 × 9–20 μm (*x̄*=230 × 12 μm, n = 20), macronematous, simple, solitary, branched at the apex, stipes straight or flexuous, cylindrical, erect, septate, smooth, dark brown to reddish brown, 8–14 septa, brown, well-defined small pores with dark scar at the apex, smooth or verruculose. ***Conidiogenous cells*** 17–60 × 9–12 μm (*x̄*=40 × 12 μm, n = 10), monotretic or polytretic, integrated, terminal, becoming intercalary, doliiform to oblong, pale brown. ***Conidia*** 63–142 × 16–26 μm (*x̄* = 87 × 20 μm, n = 20), phragmosporous, acrogenous, broad fusiform or obclavate, distoseptate at the early state, 3–12-euseptate at maturity, slightly constricted at septa, dark brown to reddish brown, verrucose, gradually tapering to 13 μm at the distal end, with a dark brown to black scar present at the base, subhyaline, elongate cells at the upper end of conidia, with (1–)2 guttules in each cell.

##### Material examined.

Thailand, • Chiang Rai Province, Mae Sai District, dead stems of *Clematis sikkimensis*, 24 June 2017, C. Phukhamsakda & *M. van* de Bult, CMTHDT02 (MFLU 17–1494, holotype); ex-type living culture, MFLUCC 17–2086.

##### Notes.

We have reverified the type specimen of *Pseudohelminthosporium clematidis* and reinstated the genus *Pseudohelminthosporium*. Koukol and Delgado (2021) treated *Pseudohelminthosporium* as a synonym of *Neopodoconis*, considering *P. clematidis* conspecific with *N. ampullacea*. However, we do not concur with this taxonomic interpretation as their conclusion was largely based on morphological resemblance without any molecular support. The morphological characteristics of *Pseudohelminthosporium*, particularly its mode of conidiogenesis, conidial morphology, and stromatic development, are sufficiently distinct from *Neopodoconis*. While Koukol and Delgado (2021) emphasised the similarity in polytretic, sympodial conidiogenesis and conidial septation patterns, their argument fails to acknowledge the variability and structural nuances described in the protologue of *P. clematidis* by Phukhamsakda et al. (2020). Moreover, the synonymy was proposed without molecular data derived from the type materials of *P. clematidis* and *N. ampullacea*, rendering their conclusion speculative. Therefore, until comparative molecular data from authentic type specimens of both taxa are available, the synonymy remains inconclusive. Based on morphological and developmental features, we uphold *Pseudohelminthosporium* as a distinct and valid genus within *Neomassarinaceae*, based on multigene phylogenetic analyses. The same applies to the original publication by Phukhamsakda et al. (2020), which placed this genus within *Neomassarinaceae*. Notably, the description provided by Phukhamsakda et al. (2020) has been revised following a re-examination of the type specimen. A discrepancy was noted between the conidial septation mentioned in the original description and illustrated conidia (fig. 37j, p. 69). Based on this re-evaluation, the conidial septation is amended to 3–12-euseptate, rather than 3–5-euseptate as originally reported.

A detailed synopsis table of morphologically similar genera *Neopodoconis*, *Pseudohelminthosporium*, *Rostriconidium*, and *Sporidesmioides* is given in Table 3.

**Table 3.** A comparison table of *Neopodoconis*, *Pseudohelminthosporium*, *Rostriconidium*, and *Sporidesmioides*.

**Genus**	**Conidiophores**	**Conidiogenous cells**	**Conidia**	**Conidiogenesis**	**Family/Phylogetic position**	**References**
*Neopodoconis* Rifai	Macronematous, mononematous, erect, straight to flexuous, unbranched, brown, smooth, septate.	Polytretic, integrated, terminal becoming intercalary, sympodial, cicatrized.	Acropleurogenous, obclavate to broadly obclavate, rostrate, euseptate, smooth to verruculose, brown, paler at apex, with truncate basal scar.	Holoblastic, tretic (polytretic), sympodial proliferation.	*Incertae sedis*	Rifai (2008)
*Pseudohelminthosporium* Phukhams. & K.D. Hyde	Macronematous, solitary, simple, branched at apex, cylindrical, straight to flexuous, dark brown to reddish brown, multi-septate, smooth to verruculose, with apical pores.	Monotretic or polytretic, integrated, terminal, doliiform to oblong, pale brown.	Acrogenous, fusiform to obclavate, phragmosporous, distoseptate when young becoming euseptate at maturity, verrucose, dark brown to reddish brown, with basal scar and elongated hyaline apical cells.	Tretic (mono-/polytretic), via apical pores.	* Neomassarinaceae *	Phukhamsakda et al. (2020)
*Rostriconidium* Z.L. Luo, K.D. Hyde & Hong Y. Su	Macronematous, mononematous, solitary, erect, straight to flexuous, smooth-walled, septate, dark brown to black, apex paler	Monotretic or polytretic, integrated, terminal, cylindrical, dark brown.	Solitary, dry, pyriform when young becoming rostrate at maturity, dark brown to black, smooth, with thick truncate basal scar and pale cell above, apex narrowly cylindrical to obtuse.	Tretic (mono-/polytretic)	* Torulaceae *	Su et al. (2018)
*Sporidesmioides* Jun F. Li, Phookamsak & K.D. Hyde	Macronematous, mononematous, erect, straight or curved, unbranched, cylindrical, smooth, thick-walled, brown to dark brown, arising from stromatic base	Polyblastic, integrated, terminal, sometimes intercalary, sympodial or percurrent, cylindrical to doliiform.	Acrogenous, solitary, pyriform to fusiform or obclavate, rostrate, septate, smooth to verruculose or echinulate, brown to dark brown, paler at apex, truncate at base.	Holoblastic, polyblastic, sympodial or percurrent; conidial secession schizolytic.	* Sporidesmioidesaceae *	This study

## Discussion

Presently, *Rostriconidium* comprises five phylogenetically accepted species. All previously known *Rostriconidium* species have been described from the Yunnan Province of China and were isolated from decaying plant litter from freshwater and terrestrial habitats (Li et al. 2016; Su et al. 2018; Tibpromma et al. 2018; Shen et al. 2021; Wang et al. 2023). Our new species, *R. synnematicum*, also represents the first report of *Rostriconidium* from the terrestrial habitat of the Western Ghats, India. Phylogenetically, *R. synnematicum*AMH 10845 formed a separate clade within *Rostriconidium (Torulaceae)* with strong statistical support (ML-BS = 100%/BY-PP = 1.00) (Fig. 2), basal to the remaining species in *Rostriconidium*. *Rostriconidium synnematicum*, with a conidial size of 44–70 × 13–22 μm and 4–6 septa, is the *Rostriconidium* species with the smallest conidia reported (Table 2). All *Rostriconidium* species are characterised by dark brown to pale brown conidia with a paler apex and a fusiform to pyriform shape. Mature conidia of *R. synnematicum* are of typical *Rostriconidium* conidia, but conidial shape is obpyriform at a young age. *Rostriconidium synnematicum* is the only synnematous species of *Rostriconidium*, while the rest are characterised by macro or mononematous conidiophores.

Furthermore, we have identified an anomaly in the descriptions of *Rostriconidium cangshanense*, *R. pandanicola*, and *R. yunnanensis* pertaining to their conidiogenesis. Although the published photoplates clearly show enteroblastic, monotretic, or polytretic conidiogenesis, the original descriptions incorrectly referred to it as monoblastic or polyblastic.

This inconsistency may lead to confusion, and we have included this change in conidiogenesis in the comparison table (Table 2) for each species that needs it. As per the generic description of *Rostriconidium*, conidiogenesis should correctly be characterised as mono or polytretic. We also propose synonymising *Neopodoconis yunnanensis*, introduced by Wang et al. (2023), under *Rostriconidium*, as it forms an independent lineage within the genus in our phylogenetic analyses. Wang et al. (2023) introduced a species under *Neopodoconis* following the preprint paper by Qiu et al. (2022).

This study proposes a new family, *Sporidesmioidesaceae*, to accommodate *Sporidesmioides*, a monotypic genus established by Li et al. (2016) from Thailand. Currently, *Pleosporales* comprises 95 families, including our newly proposed family, in which two families lack molecular data. This study was mainly conducted to resolve the existing taxonomic chaos in the phylogenetic placement of *Rostriconidium* and *Sporidesmioides* as well as the familial placement and classification of *Neopodoconis*-*Rostriconidium*-*Sporidesmioides* species complex. Upon comparing the morphological characters of species currently placed in *Rostriconidium* and *Sporidesmioides*, we conclude that morphology alone is insufficient for reliable delimitation at the generic or species level due to significant character overlap. We have also reinstated the genus *Pseudohelminthosporium* (Phukhamsakda et al. 2020) rather than synonymising it under *Neopodoconis* solely based on morphological evidence presented by Koukol and Delgado (2021). Here, we emphasise the importance of molecular tools-based species recognition and phylogenetic placement, as well as synonymisation at the generic level based on DNA sequences from type material. According to our current LSU-ITS-SSU-*tef1α*-*rpb2*-based phylogenetic analyses, *Rostriconidium*, and *Sporidesmioides* are placed in *Torulaceae*, and *Sporidesmioidesaceae*, respectively. *Pseudohelminthosporium*, another morphologically similar and cryptic species under *Neomassarinaceae*.

Phylogenetically, our new family, *Sporidesmioidesaceae*, is related to three members of the *Pleosporales*: *Torulaceae*, *Roussoellaceae*, and *Thyridariaceae. Sporidesmioidesaceae*, accommodating the monotypic genus *Sporidesmioides*, is restricted to Thailand and isolated from the stem of a dead herb (Li et al. 2016). Morphologically, *Sporidesmioidesaceae* resembles *Torulaceae* in having only asexual morphs reported. Species of *Torulaceae* have been naturally found as saprobes on a wide range of hosts in both aquatic and terrestrial habitats, of which most species were isolated from submerged wood, dead branches, and herbaceous litter of various vascular plants worldwide, such as in plant families and reported as opportunistic pathogens to both plants and humans (Crous et al. 2015; Su et al. 2016; Li et al. 2017; Li YX et al. 2023). Ecologically, *Roussoellaceae* and *Thyridariaceae* predominantly inhabit woody substrates, including bamboo, palms, and other plant hosts, functioning as saprobes, though *Roussoellaceae* has been reported as a human pathogen on rare occasions (Hyde et al. 2013; Liu et al. 2014; Phookamsak et al. 2019). *Thyridariaceae* members also inhabit marine and semi-marine environments, exemplified by the genus *Thyridariella*, highlighting a broader ecological range than *Roussoellaceae* (Jaklitsch and Voglmayr 2016; Devadatha et al. 2018). *Roussoellaceae* and *Thyridariaceae* are closely related families within the order *Pleosporales (Dothideomycetes)*, and their phylogenetic relationships have been clarified through multi-gene analyses including ITS, LSU, SSU, *tef1*-*α*, *rpb2*, and *tub2* sequences (Jaklitsch and Voglmayr 2016; Phookamsak et al. 2019). *Roussoellaceae*, established by Liu et al. (2014), is well-resolved with a monophyletic lineage supported by both morphological and molecular data, and currently comprises 12 genera (Hyde et al. 2024). The family was historically conflated with *Thyridariaceae* due to morphological similarities, but phylogenetic studies demonstrate that *Roussoellaceae* forms a distinct clade separate from *Thyridariaceae*, despite sharing bitunicate asci, immersed ascomata, and coelomycetous asexual morphs. *Thyridariaceae*, introduced by Hyde et al. (2013), initially included *Thyridaria* and related genera that had previously been variably placed in *Didymosphaeriaceae*, *Lophiostomataceae*, *Melanommataceae*, *Platystomaceae*, *Pleosporaceae*, and *Sphaeriaceae*. Molecular phylogenetic analyses (Schoch et al. 2009; Jaklitsch and Voglmayr 2016; Phookamsak et al. 2019) confirmed *Thyridariaceae* as a distinct, well-supported family within *Pleosporales*. Currently, it includes nine genera: *Chromolaenomyces*, *Cycasicola*, *Liua*, *Parathyridaria*, *Parathyridariella*, *Pseudothyridariella*, *Thyridaria*, *Thyridariella* and *Vaginospora* (Hyde et al. 2024). Recent studies have refined the placement of certain genera formerly assigned to *Roussoellaceae*, such as *Neoconiothyrium* and *Pararoussoella* (Li WL et al. 2023). *Sporidesmioidesaceae* is a new lineage introduced in this study as basal to *Roussoellaceae*.

We do not accept the conclusions of Koukol and Delgado (2021), who synonymised *Pseudohelminthosporium* (Phukhamsakda et al. 2020) with *Neopodoconis ampullacea* and placed it within *Neomassarinaceae*. Rather, we reinstate the *Pseudohelminthosporium*; however, we acknowledge that additional sequence data and robust phylogenetic analyses are required to confirm the congeneric status of these two genera. This study highlights the indispensable role of molecular data in species delimitation, particularly when morphological characters are ambiguous or overlapping. Comparative analysis of morphological traits across all known species of *Rostriconidium*, *Neopodoconis*, and *Sporidesmioides*, illustrating the limitations of morphology alone in resolving generic boundaries.

In this study, we do not recognise the five species introduced by Qiu et al. (2022) as valid, as they were not published in accordance with the rules of the International Code of Nomenclature for algae, fungi, and plants (ICNafp), and their details are not available in either Index Fungorum or MycoBank. As per *ICNafp*, specifically, Articles 29.1 and 32.1(c), mandate effective publication in a journal with an ISSN and mandatory registration of fungal names in recognised repositories for valid publication. In their preprint paper, Qiu et al. (2022) synonymised *Rostriconidium* and *Sporidesmioides* under *Neopodoconis* based on morphological similarities. But their treatment also lacked molecular evidence from the type material of *Neopodoconis ampullacea*. According to Index Fungorum, the currently accepted species under *Neopodoconis* include *N. ampullacea* (Petch) Rifai 2008 (the type species), *N. gymnemae* (P.N. Singh and S.K. Singh) R.F. Castañeda and D.W. Li 2024, *N. megasperma* (Boedijn) Rifai 2008, *N. minigelatinosa* (Aramb., Cabello and Mengasc.) R.F. Castañeda and D.W. Li 2024, and *N. yunnanensis* W.P. Wang, H.W. Shen and Z.L. Luo 2023. In our study, *N. yunnanensis* is synonymised under *Rostriconidium* based on multigene phylogenetic analyses. *Neopodoconis gymnemae*, *N. megasperma*, and *N. minigelatinosa* were introduced under *Neopodoconis* solely on morphological grounds. Considering the morphological overlap within the *Neopodoconis-Rostriconidium-Sporidesmioides-Pseudohelminthosporium* species complex, its taxonomic position must be confirmed through phylogenetic analyses once sequence data become available. Insufficient sequence data for species-level resolution further weakens the phylogenetic analyses presented by Qiu et al. (2022), which are based on LSU and SSU sequence data. Our study emphasises the need for comprehensive literature reviews to avoid taxonomic misplacements. It underscores the importance of prioritising molecular evidence over morphology for accurate and reliable species identification and stable classification.

This study also reassessed *Megacapitulaceae* (Rajeshkumar et al. 2024) under *Pleosporales*. The GenBank accession numbers were inadvertently omitted in the original publication in Kavaka. The original sequences used to validate the phylogenetic position of *Megacapitula villosa* under *Megacapitulaceae* are included in Suppl. material 2.

Although this study provides a revised taxonomic framework for *Rostriconidium* and related genera, several limitations remain. First, molecular data were limited for certain taxa, and some targeted gene regions could not be amplified across all specimens, potentially reducing phylogenetic resolution in specific clades. For example, repeated attempts to amplify the internal transcribed spacer (ITS) region of the nuclear ribosomal DNA (ITS1, 5.8S, ITS2) in *Rostriconidium synnematicum* were unsuccessful, preventing the inclusion of this locus in the multigene dataset for this species. Second, sexual morphs were unavailable for most taxa, which restricted interpretation of life cycle characteristics and limited comparative analyses based on reproductive structures. Third, the study relied primarily on collections from a restricted geographical area, particularly the Western Ghats, and on sequence data from public repositories, which may not represent the full global diversity and distribution of these lineages. Furthermore, several taxa examined belong to historically complex or poorly resolved species complexes, where type material was either inaccessible or not re-sequenced, thereby limiting definitive taxonomic resolution in some instances.

Future research should prioritise expanding both geographical and ecological sampling to more accurately represent the diversity of these fungal groups across various biogeographic regions. The inclusion of fresh collections linked to type or epitype material, combined with successful sequencing of multiple loci from type specimens when feasible, will be essential for resolving outstanding taxonomic ambiguities. Incorporating sexual morph data, when available, will enhance morphological interpretations and facilitate species delimitation. Additionally, studies utilising genome-scale data and phylogenomic methods are strongly recommended to clarify deeper evolutionary relationships within Pleosporales and related lineages, especially among morphologically convergent and polyphyletic genera. These integrative approaches will further refine the taxonomy and evolutionary understanding of these complex fungal groups.

## Supplementary Material

XML Treatment for Sporidesmioidesaceae

XML Treatment for Rostriconidium synnematicum

XML Treatment for Rostriconidium yunnanensis

XML Treatment for Pseudohelminthosporium

XML Treatment for Pseudohelminthosporium clematidis

## Additional information

### Artificial Intelligence (AI) use

The authors accept full responsibility for the content of the manuscript, including the disclosure of any use of AI.

No AI tools were used in the preparation of this manuscript.

### Author ORCIDs

S. O. Paraparath https://orcid.org/0009-0001-6984-5947

K. C. Rajeshkumar https://orcid.org/0000-0003-0401-8294

S. Siddiqui https://orcid.org/0000-0001-9429-887X

D. Chandran https://orcid.org/0009-0008-2789-2180

R. Phookamasak https://orcid.org/0000-0002-6321-8416

J. Li https://orcid.org/0009-0008-6088-2072

N. N. Wijayawardene https://orcid.org/0000-0003-0522-5498

R. K. Verma https://orcid.org/0000-0002-1790-9195

S. C. Karunarathna https://orcid.org/0000-0001-7080-0781

S. Tibpromma https://orcid.org/0000-0002-4706-6547

A. K. Gautam https://orcid.org/0000-0003-0140-7710

R. Kapoor https://orcid.org/0000-0002-7817-2641

N. Suwannarach https://orcid.org/0000-0002-2653-1913

R. Jeewon https://orcid.org/0000-0002-8563-957X

### Data availability

All of the data that support the findings of this study are available in the main text or Supplementary Information.

## Supplementary materials

Supplementary material 1 Maximum Likelihood (ML) phylogenetic tree inferred from an IQ-TREE analysis of representative species in 93 families of *Pleosporales* based on combined LSU, ITS, SSU, *tef1*-*α*, and *rpb2* sequence data Data type pdf Explanation note Branch support values are shown at the nodes in the order: ML bootstrap support (ML-BS) / gene concordance factor (gCF) / site concordance factor (sCF). ML bootstrap values were calculated from 1,000 non-parametric replicates. The tree is rooted with members of *Berkleasmium (Tubeufiales)*. https://binary.pensoft.net/file/1731353This dataset is made available under the Open Database License (http://opendatacommons.org/licenses/odbl/1.0/). The Open Database License (ODbL) is a license agreement intended to allow users to freely share, modify, and use this Dataset while maintaining this same freedom for others, provided that the original source and author(s) are credited. Sruthi O. Paraparath, Kunhiraman C. Rajeshkumar, Shadma Siddiqui, Dilna Chandran, Rungtiwa Phookamasak, Junfu Li, Nalin N. Wijayawardene, Rajnish Kumar Verma, Samantha C. Karunarathna, Sawoluck Tibpromma, Ajay Kumar Gautam, Rupam Kapoor, Nakarin Suwannarach, Rajesh Jeewon

Supplementary material 2 Taxa and their corresponding GenBank accession numbers used in this study (newly generated sequences are indicated in bold and – indicates unavailable data). Data type docx https://binary.pensoft.net/file/1731354This dataset is made available under the Open Database License (http://opendatacommons.org/licenses/odbl/1.0/). The Open Database License (ODbL) is a license agreement intended to allow users to freely share, modify, and use this Dataset while maintaining this same freedom for others, provided that the original source and author(s) are credited. Sruthi O. Paraparath, Kunhiraman C. Rajeshkumar, Shadma Siddiqui, Dilna Chandran, Rungtiwa Phookamasak, Junfu Li, Nalin N. Wijayawardene, Rajnish Kumar Verma, Samantha C. Karunarathna, Sawoluck Tibpromma, Ajay Kumar Gautam, Rupam Kapoor, Nakarin Suwannarach, Rajesh Jeewon
