# Supplementary material for: Phenotypic plasticity and cryptic generic enigma in Pleosporales: Establishing Sporidesmioidesaceae fam. nov. and Rostriconidium synnematicum sp. nov. from India
Source: MycoKeys. 2026 Jul 30;138:1–33. doi: 10.3897/mycokeys.138.184414 (PMC13448397; doi:10.3897/mycokeys.138.184414)
Supplement: Supplementary file 1 [file mycokeys-138-001-s002.docx]

Supplementary files

TABLE S1. Taxa and their corresponding GenBank accession numbers used in this study (newly generated sequences are indicated in bold and – indicates unavailable data).

| **Species** | **Isolate/strain number** | **LSU** | **ITS** | **SSU** | ***tef*1-α** | ***rpb*2** |
| --- | --- | --- | --- | --- | --- | --- |
| *Acrocalymma aquatica* | MFLUCC 11-0208 ^T^ | JX276952 | JX276951 | JX276953 | – | – |
| *Acrocalymma pterocarpi* | MFLUCC 17 0926 ^T^ | MK347949 | MK347732 | MK347840 | MK360040 | – |
| *Ageratinicola kunmingensis* | KUMCC 21-0217 ^T^ | NG_243113 | NR_191196 | NG_242816 | – | – |
| *Aigialus grandis* | BCC 20000 ^T^ | GU479775 | – | GU479739 | GU479839 | – |
| *Alternaria atrobrunnea* | FMR 16868 ^T^ | – | LR537033 | – | LR537051 | LR537044 |
| *Alternaria alternata* | AFTOL-ID 1610 ^T^ | DQ678082 | KF465761 | KC584507 | KC584634 | KC584375 |
| *Amniculicola aquatica* | MFLUCC 16-1123 ^T^ | MK106096 | – | MK106108 | MK109800 | – |
| *Amorocoelophoma cassiae* | MFLUCC 17-2283 | MK347956 | MK347739 | NG_065775 | MK360041 | MK434894 |
| *Anastomitrabeculia didymospora* | MFLU 20-0694 ^T^ | MW412978 | NR_172008 | NG_073568 | MW411338 | – |
| *Anastomitrabeculia didymospora* | MFLU 11-0236 | ON077069 | ON077080 | ON077074 | ON075063 | ON075067 |
| *Angustimassarina lonicerae* | MFLUCC 15-0087 | KY496724 | KY496759 | – | – | – |
| *Anteaglonium gordoniae* | MFLUCC 17-2431 ^T^ | MK347977 | MK347761 | MK347867 | MK360042 | MK434881 |
| *Anteaglonium gordoniae* | CD7 | – | OK335788 | – | – | – |
| *Anteaglonium latirostrum* | GKM1119 ^T^ | GQ221874 | – | – | GQ221937 | – |
| *Anteaglonium latirostrum* | GKML100Nb | GQ221876 | – | – | GQ221938 | – |
| *Aquadictyospora clematidis* | MFLUCC 17-2080 ^T^ | MT214545 | MT310592 | MT226664 | MT394727 | MT394679 |
| *Aquastroma magniostiolata* | HHUF 30122 ^T^ | AB807510 | LC014540 | AB797220 | AB808486 | – |
| *Aquasubmersa japonica* | HHUF 30469 ^T^ | NG_057138 | NR_154739 | NG_062426 | LC194384 | LC194421 |
| *Aquasubmersa japonica* | MFLUCC 17-2121 | OP377971 | OP377885 | OP378047 | OP473059 | OP473118 |
| *Aquasubmersa japonica* | MFLUCC 15-0622 | OP377958 | OP377872 | OP378036 | OP473051 | OP473112 |
| *Aquasubmersa mircensis* | MFLUCC 11-0401 ^T^ | NG_042699 | JX276954 | NG_061141 | – | – |
| *Aquihelicascus songkhlaensis* | MFLUCC 18-1154 ^T^ | MN913692 | MT627680 | – | MT954380 | – |
| *Aquihelicascus songkhlaensis* | MFLUCC 18-1273 | MN913724 | MT627696 | MT864319 | MT954369 | MT878464 |
| *Aquimassariosphaeria kunmingensis* | KUMCC 18-1019 ^T^ | MT627661 | – | MT864312 | MT954409 | MT878454 |
| *Ascocylindrica marina* | MD6011 ^T^ | KT252905 | – | KT252907 | – | – |
| *Ascocylindrica marina* | MF416 | MK007123 | – | MK007124 | – | – |
| *Astragalicola vasilyevae* | MFLUCC 17-0832 ^T^ | MG828986 | NR_157504 | MG829098 | MG829193 | MG829248 |
| *Astrosphaeriella fusispora* | MFLUCC 10-0555 | KT955462 | – | KT955443 | KT955425 | KT955413 |
| *Atrocalyx glutinosus* | DAOM: 252609 ^T^ | OQ400928 | OQ400918 | – | OQ413076 | OQ413081 |
| *Atrocalyx glutinosus* | CHEM 2721 | OQ400930 | OQ400920 | – | – | OQ413084 |
| *Bahusandhika indica* | GUFCC 18001 | KF460274 | KF460273 | – | – | – |
| *Bambusicola bambusae* | MFLUCC 11-0614 ^T^ | JX442035 | JX442031 | JX442039 | – | KP761718 |
| *Berkleasmium aquaticum* | MFLUCC 17-0049 ^T^ | KY790432 | KY790444 | – | KY792608 | MF535268 |
| *Berkleasmium aquaticum* | MFLUCC 17-0039 | KY790431 | KY790443 | – | KY792607 | MF535267 |
| *Berkleasmium longisporum* | MFLUCC 17-1999 ^T^ | MH558825 | MH558698 | – | MH550889 | MH551012 |
| *Berkleasmium longisporum* | MFLUCC 17-2002 | MH558826 | MH558699 | – | MH550890 | MH551013 |
| *Bertiella fici* | MFLU 19-2713 ^T^ | MW063223 | – | MW079351 | MW183786 | – |
| *Bertiella fici* | NCYU 19-0073 | MW063224 | – | MW079352 | MW183787 | – |
| *Bipolaris adikaramae* | HSF070 ^T^ | – | MN535176 | – | MT548605 | – |
| *Boeremia linicola* | CBS 116.76 ^T^ | GU237938 | GU237754 | – | KY484705 | KT389574 |
| *Boeremia linicola* | CBS 248.38 | KT389703 | KT389486 | – | – | KT389575 |
| *Biatriospora borsei* | NFCCI-4245 ^T^ | MK358813 | MK358818 | MK358811 | MK330938 | – |
| *Biatriospora marina* | CY 1228 | GQ925848 | – | GQ925835 | GU479848 | GU479823 |
| *Brevicollum hyalosporum* | MAFF 243400 ^T^ | LC271239 | LC271242 | LC271236 | LC271245 | LC271249 |
| *Brevicollum hyalosporum* | MFLUCC 17-0071 | MG602200 | MG602204 | MG602202 | MG739516 | – |
| *Brevicollum versicolor* | HHUF 30591 ^T^ | NG_058716 | NR_156335 | NG_065124 | LC271246 | LC271250 |
| *Brunneoclavispora camporesii* | MFLUCC 11-0001 ^T^ | MN809328 | MN809329 | – | – | – |
| *Brunneofusispora clematidis* | MFLUCC 17-2070 ^T^ | MT214570 | MT310615 | MT226685 | MT394629 | MT394692 |
| *Camarosporium quaternatum* | CPC 31081 ^T^ | NG_064442 | NR_159756 | KY929123 | KY929201 | – |
| *Camarosporomyces flavigenus* | CBS 314.80 ^T^ | GU238076 | MH861266 | NG_061093 | – | – |
| *Camarosporidiella caraganicola* | MFLUCCC 14-0605 ^T^ | KP711381 | KP711380 | KP711382 | – | – |
| *Camarosporidiella melnikii* | MFLUCC 17-0684 ^T^ | MF434250 | MF434162 | MF434338 | MF434425 | – |
| *Capulatispora sagittiformis* | HHUF 29754 ^T^ | NG_042319 | NR_119393 | NG_060997 | LC001756 | – |
| *Caryospora aquatica* | MFLU 11-1083 ^T^ | NG_059058 | NR_156408 | MH057850 | – | – |
| *Caryospora submersa* | MFLUCC 18-1283 ^T^ | MN913720 | – | – | – | – |
| *Clematidis italica* | MFLUCC 15-0084 ^T^ | KU842381 | KU842380 | KU842382 | – | – |
| *Coelodictyosporium rosarum* | MFLUCC 17-0776 ^T^ | MG828991 | MG828875 | MG829102 | MG829195 | – |
| *Corylicola italica* | MFLU 19-0500 ^T^ | MT554926 | MT554925 | MT554923 | – | MT590776 |
| *Corynespora cassiicola* | CBS 100822 | GU301808 | – | GU296144 | GU349052 | GU371742 |
| *Corynespora torulosa* | CPC 15989 ^T^ | KF777207 | NR_145181 | – | – | – |
| *Crassimassarina macrospora* | MAFF 239606 ^T^ | LC194344 | LC194478 | LC194302 | LC194389 | LC194426 |
| *Crassimassarina macrospora* | HHUF 30512 | LC194343 | LC194477 | LC194301 | LC194388 | LC194425 |
| *Crassiperidium octosporum* | MAFF 242971 ^T^ | LC373108 | LC373096 | LC373084 | LC373120 | LC373132 |
| *Crassiperidium octosporum* | MAFF 246401 | LC373111 | LC373099 | LC373087 | LC373123 | LC373135 |
| *Cryptocoryneum japonicum* | HHUF 30482 ^T^ | NG_059035 | NR_153938 | NG_065118 | LC096144 | LC194438 |
| *Cryptocoryneum pseudorilstonei* | CBS 113641 ^T^ | NG_059036 | NR_153941 | LC194322 | LC096152 | LC194446 |
| *Cucurbitaria berberidis* | MFLUCC 11-0387 | KC506796 | – | KC506800 | – | – |
| *Curvularia austriaca* | CBS 102694 ^T^ | – | MN688802 | – | MN688856 | – |
| *Curvularia eleusinicola* | USJCC-0005 ^T^ | – | MT262877 | – | MT432925 | – |
| *Cylindroaseptospora leucaenicola* | MFLUCC 17-2424 ^T^ | MK347966 | NR_163333 | MK347856 | – | – |
| *Cyclothyriella rubronotata* | CBS 141486 ^T^ | KX650544 | NR_147651 | NG_061252 | KX650519 | KX650574 |
| *Cylindrotorula indica* | NFCCI 4836^T^ | NG_081308 | NR_175156 | - | MT321492 | MT321490 |
| *Cylindrotorula indica* | NFCCI 4837^T^ | MT339443 | MT339445 | - | MT321493 | MT321491 |
| *Dacampia hookeri* | GZU 73897 | KT383792 | – | – | – | – |
| *Dacampia hookeri* | GZU 81840 | KT383795 | – | – | – | – |
| *Dacampia hookeri* | GZU 75980 | KT383794 | – | – | – | – |
| *Delitschia nypae* | MFLUCC 17-2588 ^T^ | – | – | – | MK360049 | MK434878 |
| *Dendryphion aquaticum* | MFLUCC 15-0257^T^ | KU500573 | KU500566 | KU500580 | – | – |
| *Dendryphion comosum* | CBS 208.69 | MH871026 | MH859293 | – | – | – |
| *Dendryphion europaeum* | CBS:137991 | MH877652 | NR_158390 | – | – | – |
| *Dendryphion fluminicola* | MFLUCC 17-1689 ^T^ | MG208141 | NR_157490 | – | MG207992 | – |
| *Dendryphion europaeum* | CBS:137991 | MH877652 | NR_158390 | – | – | – |
| *Dendryphion hydei* | KUN-HKAS:112706 | MW879527 | MW723060 | MW774583 | – | – |
| *Dendryphion submersum* | DLUCC 0698 | MG208137 | MG208158 | – | MG207988 | MG207969 |
| *Deniquelata cassiae* | CMD012A ^T^ | OR500088 | OR500092 | OR500090 | OR501827 | – |
| *Dictyocheirospora bannica* | KH 332 ^T^ | AB807513 | LC014543 | AB797223 | AB808489 | – |
| *Didymella exigua* | CBS 183.55 ^T^ | MH868977 | MH857436 | GU296147 | – | – |
| *Didymella rumicicola* | CBS 683.79 ^T^ | MH873007 | KT389503 | – | – | KT389622 |
| *Dothidotthia robiniae* | MFLUCC 16-1175 ^T^ | MK751817 | MK751727 | MK751762 | MK908017 | MK920237 |
| *Epicoccum duchesneae* | CGMCC 3.18345 ^T^ | KY742249 | KY742095 | – | – | MT018115 |
| *Epicoccum duchesneae* | CBS 218.81 | MN973322 | MN972935 | – | – | MN983572 |
| *Falciformispora aquatica* | MFLUCC 18-0212 ^T^ | MK063643 | MK064216 | – | MK099811 | – |
| *Falciformispora tompkinsii* | CBS 200.79 ^T^ | MH872968 | MH861199 | KF015639 | KF015685 | KF015719 |
| *Fenestella crataegi* | CBS 144857 ^T^ | – | NR_165534 | – | MK357555 | MK357512 |
| *Fissuroma calami* | MFLUCC 13-0836 ^T^ | MF588993 | – | NG_062430 | MF588975 | – |
| *Flammeascoma lignicola* | MFLUCC 10-0128 ^T^ | KT324583 | KT324582 | KT324584 | KT324585 | KT324586 |
| *Flavomyces fulophazii* | CBS 135761 ^T^ | NG_058131 | NR_137960 | NG_061191 | – | – |
| *Foliophoma fallens* | CBS 161.78 | GU238074 | KY940772 | GU238215 | – | KC584502 |
| *Fuscostagonospora cytisi* | MFLUCC 16-0622 ^T^ | KY770978 | – | KY770977 | KY770979 | – |
| *Fuscostagonospora sasae* | HHUF 29106 ^T^ | AB807548 | AB809636 | AB797258 | AB808524 | – |
| *Fusculina eucalypti* | CBS 120083 ^T^ | DQ923531 | DQ923531 | – | – | – |
| *Fusculina eucalyptorum* | CBS 145083 ^T^ | MK047499 | NR_161140 | – | – | – |
| *Fusiformispora clematidis* | MFLUCC 17-2077 ^T^ | MT214542 | MT310589 | MT226661 | MT394725 | MT394677 |
| *Gordonomyces mucovaginatus* | CMW 22212 ^T^ | JN712552 | JN712486 | – | – | – |
| *Halojulella avicenniae* | BCC 20173 ^T^ | GU371822 | – | GU371830 | GU371815 | GU371786 |
| *Helminthosporiella stilbacea* | MFLUCC 15-0813 ^T^ | MT928157 | MT928159 | MT928161 | MT928151 | – |
| *Hermatomyces clematidis* | MFLUCC 17-2085 ^T^ | MT214556 | MT310603 | MT226673 | MT394735 | MT394684 |
| *Hermatomyces trangensis* | BCC 80741 ^T^ | KY790600 | KY790598 | KY790602 | KY790606 | KY790604 |
| *Hermatomyces trangensis* | BCC 80742 | KY790601 | KY790599 | KY790603 | KY790607 | KY790605 |
| *Hongkongmyces aquaticus* | MFLUCC 18-1150 ^T^ | MN913694 | – | MT864302 | MT954379 | – |
| *Hypsostroma caimitalense* | GKM1165 ^T^ | GU385180 | – | – | – | – |
| *Hypsostroma thailandicum* | MFLUCC 21-0057 ^T^ | MZ435867 | MZ435865 | – | – | – |
| *Jeremyomyces labinae* | CBS 144617 ^T^ | MK442529 | MK442589 | – | MK442695 | MK442665 |
| *Juncaceicola alpina* | CBS 456.84 ^T^ | MH873460 | MH861761 | KY090699 | KF253139 | KF252188 |
| *Keissleriella camporesiana* | MFLUCC 15-0029 ^T^ | MN401741 | MN401745 | MN401743 | MN397907 | – |
| *Latorua caligans* | CBS 576.65 ^T^ | NG_058180 | – | – | – | – |
| *Latorua grootfonteinensis* | CBS 369.72 ^T^ | NG_058181 | – | – | – | – |
| *Lentimurispora urniformis* | MFLUCC 18-0497 | MH179144 | – | MH179160 | MH188055 | – |
| *Lentithecium clioninum* | HHUF 28199 ^T^ | NG_059391 | NR_154137 | NG_064845 | AB808515 | – |
| *Lentithecium pseudoclioninum* | HHUF 29055 ^T^ | NG_059392 | AB809633 | NG_064847 | AB808521 | – |
| *Leptosphaeria chatkalica* | YGS22 ^T^ | MW886099 | MW886101 | MW886100 | MW915583 | – |
| *Leptosphaerioides guizhouensis* | GZAAS 19-4017 ^T^ | OP099529 | OR225067 | OR134435 | – | – |
| *Leptosphaerioides guizhouensis* | GZAAS 19-4018 | OP099530 | OR225068 | OR134436 | – | – |
| *Leucaenicola phraeana* | MFLUCC 18-0472 ^T^ | MK348003 | MK347785 | NG_065784 | MK360060 | MK434867 |
| *Leucaenicola aseptata* | MFLUCC 17-2423 ^T^ | NG_066309 | NR_163332 | NG_065776 | MK360059 | MK434891 |
| *Libertasomyces myopori* | CPC 27354 ^T^ | NG_058241 | KX228281 | – | – | – |
| *Ligninsphaeria jonesii* | MFLUCC 15-0641 ^T^ | KU221037 | – | – | – | – |
| *Ligninsphaeria jonesii* | GZCC 15-0080 | KU221038 | – | – | – | – |
| *Lindgomyces cigarospora* | G619 ^T^ | KX655804 | KX655794 | KX655805 | – | – |
| *Lindgomyces ingoldianus* | ATCC 200398 ^T^ | AB521736 | NR_119938 | NG_016531 | – | – |
| *Longicorpus striataspora* | MFLUCC 18-0267 ^T^ | MK035988 | MK035965 | MK035973 | MK034428 | MK034436 |
| *Longiostiolum tectonae* | MFLUCC 12-0562 ^T^ | KU764700 | KU712447 | KU712459 | – | – |
| *Longipedicellata aptrootii* | MFLU 10-0297 ^T^ | KU238894 | KU238893 | KU238895 | KU238892 | KU238891 |
| *Longipedicellata megafusiformis* | SJ-KR4 ^T^ | MZ538546 | MZ538512 | – | MZ567090 | – |
| *Lonicericola qujingensis* | GMBCC1178 ^T^ | OM855602 | OM855593 | OM855616 | OM857556 | – |
| *Lophiostoma carpini* | CBS 147279 ^T^ | MW750386 | NR_173000 | – | MW752405 | MW752384 |
| *Lophiostoma clavatum* | MFLUCC 18-1316 ^T^ | MN274566 | – | MN304835 | MN328901 | – |
| *Lophiotrema eburnoides* | MAFF 242970 ^T^ | LC001707 | LC001709 | LC001706 | LC194403 | LC194458 |
| *Magnibotryascoma kunmingense* | HKAS 111919 ^T^ | MW424785 | MW424770 | MW424800 | MW430106 | MW430113 |
| *Magnibotryascoma rubriostiolatum* | CBS 140734 ^T^ | – | KU601590 | – | KU601609 | KU601599 |
| *Magnicamarosporium iriomotense* | HHUF 30125 ^T^ | AB807509 | AB809640 | AB797219 | AB808485 | – |
| *Massaria inquinans* | CBS 125591 ^T^ | MH875187 | MH863726 | HQ599442 | HQ599340 | – |
| *Massarina pandanicola* | MFLUCC 17-0596 ^T^ | MG646947 | MG646958 | MG646979 | MG646986 | – |
| *Massarioramusculicola chiangraiensis* | MFLUCC 17-2240 ^T^ | MH040228 | MH040227 | MH040229 | – | – |
| *Massariosphaeria clematidis* | MFLU 16-0174 ^T^ | MT214544 | MT310591 | MT226663 | – | – |
| *Matsushimamyces bohaniensis* | CBS 140592 ^T^ | KR350633 | KP765516 | – | – | – |
| *Megacapitula villosa* | SNC 122 | PP621037 | PP592410 | PP627300 | PP740447 | PP780225 |
| ***Megacapitula villosa*** | **NFCCI 5894** | **PQ358421** | **PQ358423** | **PQ358425** | **PQ359440** | **PQ359439** |
| *Misturatosphaeria aurantonotata* | GKM 1238 ^T^ | NG_059927 | – | – | GU327761 | – |
| *Montagnula acaciae* | MFLUCC 18-1636 ^T^ | ON117298 | ON117280 | ON117267 | ON158093 | – |
| *Montagnula aquatica* | MFLU 22-0171 ^T^ | OP605986 | OP605992 | OP600504 | – | – |
| *Montagnula aquatica* | KUNCC 23-14425 | OR583116 | OR583097 | OR583135 | OR588088 | OR588107 |
| *Morosphaeria muthupetensis* | NFCC I4219 ^T^ | MF614796 | MF614795 | MF614797 | MF614798 | – |
| *Multilocularia bambusae* | MFLUCC 11-0180 ^T^ | KU693438 | KU693446 | KU693442 | – | – |
| *Multiseptospora thailandica* | MFLUCC 11-0183 ^T^ | KP744490 | KP744447 | KP753955 | KU705657 | – |
| *Murilentithecium lonicerae* | MFLUCC 18-0675 ^T^ | MK214373 | MK214370 | MK214376 | MK214379 | – |
| *Murispora galii* | MFLUCC 13-0819 ^T^ | KT709175 | KT736081 | KT709182 | KT709189 | – |
| *Muritestudina chiangraiensis* | MFLUCC 17-2551 ^T^ | MG602248 | MG602247 | MG602249 | MG602251 | MG602250 |
| *Neoastrosphaeriella phoenicis* | MFLUCC 18-1477 ^T^ | MN712339 | MN735995 | MN699324 | MN744232 | MN744233 |
| *Neobambusicola magnoliae* | HKAS 107122 ^T^ | ON870389 | ON878076 | ON870914 | – | – |
| *Neocamarosporium goegapense* | CPC 23676 ^T^ | KJ869220 | KJ869163 | – | – | – |
| *Neocamarosporium halophilum* | RSM70 ^T^ | – | OR242722 | – | OR289930 | – |
| *Neocamarosporium halophilum* | BBB RMS57 | OR297952 | OR297950 | – | OR339881 | – |
| *Neohelicascus aquaticus* | KUMCC 19-0107 ^T^ | MT627662 | MT627719 | MT864314 | MT954384 | – |
| *Neolophiostoma pigmentatum* | MFLUCC 10-0129 ^T^ | KT324588 | KT324587 | KT324589 | KT324590 | – |
| *Neomassaria fabacearum* | MFLUCC 16-1875 ^T^ | KX524145 | – | NG_061245 | KX524149 | – |
| *Neomassaria formosana* | NTUCC 17-007 ^T^ | MH714756 | – | MH714759 | MH714762 | MH714765 |
| *Neomassaria hongheensis* | KUMCC 21-0344 ^T^ | OL423113 | OL477614 | OL423115 | OL754594 | OL754595 |
| *Neomassarina chromolaenae* | MFLUCC 17-1480 ^T^ | MT214466 | MT214372 | MT214419 | MT235785 | MT235822 |
| *Neomassarina pandanicola* | MFLUCC 16-0270 ^T^ | MG298946 | MG298945 | MG298947 | – | – |
| *Neomassarina thailandica* | MFLU 11-0144 ^T^ | NG_059718 | NR_154244 | – | – | – |
| *Neomassarina thailandica* | MFLUCC 17-1432 | MT214467 | MT214373 | MT214420 | – | – |
| *Neooccultibambusa thailandensis* | MFLUCC 16-0274 ^T^ | MH260308 | MH275074 | MH260348 | MH412780 | MH412758 |
| *Neophaeosphaeria phragmiticola* | KUMCC 16-0216 ^T^ | MG837009 | – | NG_065735 | MG838020 | – |
| *Neophaeosphaeria livistonae* | NCYUCC 19-0393 ^T^ | OQ437387 | OQ437390 | OQ437393 | – | – |
| *Neoplatysporoides aloes* | CPC 36068 ^T^ | MN567619 | NR_166316 | – | – | – |
| *Neopyrenochaeta annellidica* | MFLU 11-1105 ^T^ | MT183502 | MT185538 | – | – | – |
| *Neopyrenochaeta cercidis* | MFLUCC 18-2089 ^T^ | MK347932 | MK347718 | MK347823 | – | MK434908 |
| *Neopyrenochaeta chiangraiensis* | MFLUCC 13-0881 ^T^ | MT183503 | MT185539 | MT214975 | MT454041 | – |
| *Neopyrenochaetopsis hominis* | UTHSC: DI16-238 ^T^ | LN907381 | LT592923 | – | – | LT593061 |
| *Neoroussoella chiangmaiensis* | MFLU 22-0205 ^T^ | OQ065735 | OQ065738 | OQ065736 | OQ186448 | OQ186450 |
| *Neothyrostroma encephalarti* | CPC 35999 ^T^ | MN567613 | MN562105 | – | MN556831 | – |
| *Neotorula aquatica* | MFLUCC 15-0342 ^T^ | KU500576 | KU500569 | KU500583 | – | – |
| *Neotorula submersa* | KUMCC 15-0280 ^T^ | KX789217 | KX789214 | – | – | – |
| *Nigrograna italica* | MFLU 23-0139 ^T^ | OR538591 | OR538590 | – | OR531366 | OR531365 |
| *Occultibambusa bambusae* | MFLUCC 13-0855 ^T^ | KU863112 | KU940123 | – | KU940193 | KU940170 |
| *Occultibambusa jonesii* | GZCC 16-0117 ^T^ | KY628322 | – | KY628324 | KY814756 | KY814758 |
| *Ochraceocephala foeniculi* | CBS 145654 ^T^ | MN516774 | MN516753 | MN516743 | MN520149 | MN520145 |
| *Ohleria modesta* | CBS 141480 | – | KX650563 | KX650513 | KX650534 | KX650583 |
| *Ohleria modesta* | WU 36870 | – | KX650562 | – | KX650533 | KX650582 |
| *Omania hydei* | SQUCC 13750 ^T^ | MW077155 | MW077146 | MW077162 | MW075772 | MW276077 |
| *Paraconiothyrium kelleni* | CBS 149290 ^T^ | NG_229027 | NR_185757 | OP348926 | OP328919 | – |
| *Paradictyoarthrinium aquatica* | MFLUCC 16-1116 ^T^ | NG_064501 | NR_158861 | – | – | – |
| *Paradictyoarthrinium diffractum* | MFLUCC 13-0466 | KP744498 | KP744455 | KP753960 | – | KX437764 |
| *Paradictyocheirospora tectonae* | AMH 10301 ^T^ | MW854647 | MW854646 | – | MW854832 | – |
| *Paralophiostoma hysterioides* | PUFNI 17617 | MT912850 | MN582758 | MN582762 | – | MT926117 |
| *Paraleptosphaeria polylepidis* | MA 57843 ^T^ | – | NR_119469 | – | – | – |
| *Paraleptosphaeria polylepidis* | APA-2999 | MK795717 | MK795714 | MK795720 | MK831009 | – |
| *Paramonodictys globosa* | HKAS 129169 ^T^ | OR091331 | OR139016 | – | OR494045 | OR494048 |
| *Parapyrenochaeta protearum* | CBS 131315 | JQ044453 | JQ044434 | – | – | LT717683 |
| *Periconia delonicis* | MFLUCC 17-2584 ^T^ | NG_068611 | – | NG_065770 | – | MK434901 |
| *Periconia pseudodigitata* | KT 1395 ^T^ | AB807564 | LC014591 | AB797274 | – | – |
| *Phaeomycocentrospora xinjangensis* | CGMCC 3.20479 ^T^ | OK256190 | OK256193 | – | – | – |
| *Phaeoseptum mali* | MFLUCC 17-2108 ^T^ | MK625197 | MK659580 | – | MK647990 | MK647991 |
| *Phaeoseptum terricola* | MFLUCC 10-0102 ^T^ | MH105779 | MH105778 | MH105780 | MH105781 | MH105782 |
| *Phaeosphaeria oryzae* | CBS 110110 ^T^ | KF251689 | KF251186 | GQ387530 | – | KF252193 |
| *Pleomonodictys capensis* | CBS 968.97 ^T^ | KY853521 | MH862684 | – | – | – |
| *Pleomonodictys capensis* | DLUCC 1323 | MZ420757 | MZ420742 | – | – | MZ442696 |
| *Pleomonodictys descalsii* | FMR 12716 ^T^ | KY853522 | KY853461 | – | – | – |
| *Plenodomus changchunensis* | CCMJ 5011 ^T^ | OL897174 | OL996123 | OL984031 | – | – |
| *Plenodomus changchunensis* | CCMJ 5012 | OL966928 | OL996124 | OL984032 | – | OL944508 |
| *Pleopunctum clematidis* | MFLUCC 17-2091 ^T^ | MT214573 | MT310618 | – | MT394632 | MT394693 |
| *Pleopunctum thailandicum* | MFLUCC 21-0039 ^T^ | MZ198896 | MZ198894 | – | MZ172461 | – |
| *Polyschema sclerotigenum* | UTHSC DI14-305 ^T^ | KP769976 | KP769975 | – | – | – |
| *Prosthemium alni* | MFLUCC 17-0240 ^T^ | KY815013 | KY797636 | – | KY815019 | – |
| *Prosthemium intermedium* | HHUF 30063 ^T^ | AB553778 | AB554108 | – | – | – |
| *Pseudoasteromassaria aquatica* | MFLUCC 18-1397 ^T^ | MN913721 | MT627674 | MT864322 | MT954378 | – |
| *Pseudoastrosphaeriella longicolla* | MFLUCC 11-0171 ^T^ | KT955476 | – | – | KT955438 | KT955420 |
| *Pseudoastrosphaeriella thailandensis* | MFLUCC 11-0144 ^T^ | KT955478 | – | KT955457 | KT955440 | KT955416 |
| *Pseudoberkleasmium acaciae* | MFLUCC 17-2590 ^T^ | NG_066316 | NR_163343 | NG_065782 | MK360073 | – |
| *Pseudoberkleasmium chiangmaiense* | MFLUCC 17-1809 ^T^ | MK131260 | MK131259 | – | MK131261 | – |
| *Pseudoberkleasmium chiangmaiense* | MFLU 21-0290 | OM065940 | OM066271 | OM065948 | OM102996 | OM102997 |
| *Pseudoberkleasmium chiangmaiense* | MFLUCC 17-2088 | MT214585 | MT310630 | MT226698 | MT394643 | MT394699 |
| *Pseudoberkleasmium chiangmaiense* | DLUCC 1655 | MZ420759 | MZ420744 | MZ420749 | MZ442693 | – |
| *Pseudoberkleasmium chiangraiense* | MFLUCC 21-0154 ^T^ | OL584200 | OL584189 | OL606408 | OL912943 | OL697401 |
| *Pseudoberkleasmium chiangraiense* | MFLUCC 21-0161 | OL584201 | OL584190 | OL606409 | OL912944 | OL697402 |
| *Pseudoberkleasmium chiangraiense* | MFLUCC 21-0162 | OL584205 | OL584191 | OL606410 | OL912945 | OL697403 |
| *Pseudoberkleasmium pandanicola* | KUMCC 17-0178 ^T^ | MH260304 | MH275071 | MH260344 | – | – |
| *Pseudochaetosphaeronema chiangraiense* | MFLU 21-0083 ^T^ | MZ457922 | MZ457923 | – | MZ476770 | – |
| *Pseudochaetosphaeronema chiangraiense* | UESTCC 23.0065 | OR253260 | OR253108 | – | OR251160 | – |
| *Pseudochaetosphaeronema irregulare* | CGMCC 3.22458 ^T^ | OQ758163 | OQ798972 | OQ758194 | OQ809057 | OQ809023 |
| *Pseudochaetosphaeronema irregulare* | CGMCC 3.22461 | OQ758162 | OQ798971 | OQ758193 | OQ809056 | OQ809022 |
| *Pseudocoleodictyospora sukhothaiensis* | MFLUCC 12-0554 ^T^ | KU764710 | KU712440 | NG_062416 | – | KU712493 |
| *Pseudocoleodictyospora thailandica* | MFLUCC 12-0565 ^T^ | KU764701 | NR_154337 | NG_062417 | – | KU712494 |
| *Pseudocoleophoma heteropanacicola* | ZHKUCC 23-0880 ^T^ | OR365486 | OR365456 | – | OR700204 | – |
| *Pseudolophiotrema elymicola* | HHUF 28984 ^T^ | LC194381 | LC194505 | LC194339 | LC194418 | LC194473 |
| *Pseudomassarina clematidis* | MFLU 16-0493 ^T^ | MT214586 | MT415397 | MT226699 | MT394644 | MT394700 |
| *Pseudopyrenochaeta lycopersici* | FMR 15746 ^T^ | EU754205 | NR_103581 | NG_062728 | – | LT717680 |
| *Pseudopyrenochaeta terretris* | FMR 15327 ^T^ | LT623216 | LT623228 | – | – | LT623287 |
| *Pseudotetraploa rajmachiensis* | NFCCI 4618 ^T^ | MN937204 | MN937222 | – | – | – |
| *Pseudoxylomyces elegans* | KT 2887 | AB807598 | LC014593 | AB797308 | AB808576 | – |
| *Profundisphaeria fusiformispora* | GZAAS 20-4010 ^T^ | – | – | OR134442 | OR140432 | OR146942 |
| *Profundisphaeria fusiformispora* | GZAAS 20-4012 | OR209667 | – | OR134443 | OR140433 | – |
| *Pyrenochaetopsis leptospora* | CBS 101635 ^T^ | GQ387627 | JF740262 | NG_063097 | MF795881 | LT623282 |
| *Pyrenochaetopsis tabarestanensis* | IBRC:M 30051 ^T^ | KF803343 | NR_155636 | NG_065034 | – | – |
| *Quadricrura bicornis* | HHUF 30023 ^T^ | AB524613 | AB524797 | AB524472 | AB524828 | – |
| *Quercicola fusiformis* | MFLUCC 18-0479 ^T^ | MK348009 | MK347790 | MK347898 | MK360085 | MK434864 |
| *Quercicola guttulospora* | MFLUCC 18-0481 ^T^ | MK348010 | MK347791 | MK347899 | MK360086 | – |
| *Quixadomyces cearensis* | HUEFS 238438 ^T^ | MG970695 | NR_160606 | – | – | – |
| *Rostriconidium aquaticum* | MFLUCC 16-1113^T^ | MG208143 | MG208164 | – | MG207994 | MG207974 |
| *Rostriconidium aquaticum* | KUMCC 15-0297 | MG208144 | MG208165 | – | MG207995 | MG207975 |
| *Rostriconidium cangshanense* | MFLUCC 20-0147^T^ | MW010281 | MW010285 | – | – | MW012636 |
| *Rostriconidium pandanicola* | KUMCC 17–0176^T^ | MH260318 | MH275084 | MH260358 | MH412781 | MH412759 |
| ***Rostriconidium synnematicum*** | **NFCCI 6243** | **PX778749** | **–** | **PX778750** | **PX781545** | **PX781546** |
| *Rostriconidium yunnanensis* | KUNCC 22-10737^T^ | OP359410 | OP359401 | OP369295 | OP471613 | OP476726 |
| *Roussoella bambusarum* | GMBC 0316 ^T^ | ON479892 | ON479891 | – | ON505017 | ON505012 |
| *Roussoella guttulata* | MFLUCC 20-0102 ^T^ | MT734818 | MT734821 | – | MW022188 | MW022187 |
| *Rubroshiraia bambusae* | HKAS 102255 ^T^ | MK804658 | MK804678 | MK804704 | MK819218 | – |
| *Rubroshiraia bambusae* | HKAS 102256 | MK804659 | MK804679 | MK804705 | MK819219 | – |
| *Rutola graminis* | CBS 145906 | NG_078685 | NR_175150 | – | – | – |
| *Rutola graminis* | CPC 33695 | MN317296 | MN313815 | – | – | – |
| *Rutola kunmingensis* | HKAS124483 | OR470705 | OR470710 | OR470700 | OR753778 | OR753783 |
| *Rutola kunmingensis* | HKAS 124484 | OR470706 | OR470711 | OR470701 | OR753779 | OR753784 |
| *Rutola kunmingensis* | HKAS 124485 | OR470707 | OR470712 | OR470702 | OR753780 | OR753785 |
| *Salsuginea phoenicis* | MFLU 19-0015 ^T^ | MK405280 | – | – | MK404650 | – |
| *Salsuginea ramicola* | KT 2597.2 | GU479801 | – | GU479768 | GU479862 | GU479834 |
| *Salsuginea rhizophorae* | MFLU 18-0540 ^T^ | MN017851 | – | MN017917 | – | – |
| *Seltsamia ulmi* | CBS 143002 ^T^ | MF795794 | MF795794 | MF795794 | MF795882 | MF795836 |
| *Septoriella chlamydospora* | MFLUCC 15-0177 ^T^ | KU163654 | KU163658 | KU163655 | – | – |
| *Septoriella hibernica* | CBS 145371 ^T^ | MK540036 | MK539966 | – | – | MK540097 |
| *Setoarthopyrenia chromolaenae* | MFLUCC 17-1444 ^T^ | MT214438 | MT214344 | MT214392 | MT235768 | MT235805 |
| *Shiraia bambusicola* | GZAAS 2.0708 ^T^ | KC460982 | GQ845414 | – | – | – |
| *Shiraia bambusicola* | HKAS 102267 | MK804657 | MK804677 | MK804703 | MK819217 | MK819237 |
| *Sporidesmioides thailandica* | KUMCC 16-0012 | KX437758 | MN061348 | KX437760 | KX437767 | KX437762 |
| *Sporidesmioides thailandica* | MFLUCC 13-0840^T^ | NG_059703 | MN061347 | KX437759 | KX437766 | KX437761 |
| *Sporormurispora atraphaxidis* | MFLUCC 17-0742 ^T^ | MG829083 | MG828971 | MG829183 | – | – |
| *Sporormurispora paulsenii* | MFLUCC 17-1957 ^T^ | MK966143 | – | MK963075 | – | MN023029 |
| *Stagonospora forlicesenensis* | MFLUCC 15-0054 ^T^ | KX655547 | KX655557 | KX655552 | KX655562 | – |
| *Stagonospora imperaticola* | MFLUCC 15-0026 | KY706133 | KY706143 | KY706138 | KY706146 | KY706149 |
| *Stemphylium clematidis* | MFLUCC 14-0937 ^T^ | MT214583 | MT310628 | MT226696 | – | – |
| *Stemphylium carpobroti* | CPC 38637 ^T^ | MW175395 | MW175355 | – | – | – |
| *Striatiguttula nypae* | MFLUCC 18-0265 ^T^ | MK035992 | MK035969 | MK035977 | MK034432 | MK034440 |
| *Striatiguttula phoenicis* | MFLUCC 18-0266 ^T^ | MK035995 | MK035972 | MK035980 | MK034435 | MK034442 |
| *Subglobosporium tectonae* | MFLUCC 12-0393 ^T^ | KU764703 | KU712445 | KU712464 | – | KU712485 |
| *Subglobosporium tectonae* | MFLUCC 12-0390 | KU764702 | KU712446 | KU712463 | – | KU712495 |
| *Sublophiostoma thailandica* | MFLUCC 11-0207 ^T^ | KX534212 | MW136257 | KX534218 | KX550077 | MW088714 |
| *Sublophiostoma thailandica* | MFLUCC 11-0185 | KX534216 | MW136275 | KX534222 | KX550080 | MW088718 |
| *Submersispora variabilis* | MFLUCC 17-2360 ^T^ | MN913682 | MT627683 | MT864310 | – | – |
| *Submersispora variabilis* | N-KR15 | MZ538561 | MZ538527 | MZ538575 | MZ567103 | MZ567114 |
| *Sulcatispora acerina* | HHUF 30449 ^T^ | LC014610 | LC014597 | LC014605 | LC014615 | – |
| *Sulcatispora berchemiae* | HHUF 29097 ^T^ | AB807534 | AB809635 | AB797244 | AB808509 | – |
| *Sulcosporium thailandica* | MFLUCC 12-0004 | KT426563 | MG520958 | KT426564 | – | – |
| *Teichospora trabicola* | C134 ^T^ | KU601591 | KU601591 | – | KU601601 | KU601600 |
| *Tetraploa aquatica* | MFLU 19-0995 ^T^ | MT530452 | MT530448 | – | – | – |
| *Tetraploa aquatica* | MFLU 19-0996 | MT530453 | MT530449 | MT530454 | – | – |
| *Thyridaria acaciae* | CBS 138873 ^T^ | NG_058127 | KP004469 | – | – | – |
| *Thyridaria broussonetiae* | CBS 141481 ^T^ | KX650568 | KX650568 | KX650515 | KX650539 | KX650586 |
| *Thyrostroma jaczewskii* | MFLUCC 18-0787 ^T^ | MK765857 | MK765856 | MK765858 | – | – |
| *Torula acaciae* | CPC29737^T^ | NG_059764 | NR_155944 | – | – | KY173594 |
| *Torula aquatica* | DLUCC 0550 | MG208145 | MG208166 | – | MG207996 | MG207976 |
| *Torula aquatica* | MFLUCC 16-1115^T^ | MG208146 | MG208167 | – | – | MG207977 |
| *Torula aquilariae* | KUNCC 24-18640^T^ | PQ788524 | PQ788522 | – | PQ810572 | PQ810570 |
| *Torula aquilariae* | HKAS 145332^T^ | PQ788523 | PQ788521 | – | PQ810571 | PQ810569 |
| *Torula breviconidiophora* | KUMCC18-0130^T^ | MK071672 | MK071670 | MK071697 | MK077673 | – |
| *Torula calceiformis* | HKAS 125551^T^ | OP751052 | OP751054 | OP751050 | OQ630512 | OQ630510 |
| *Torula calceiformis* | HKAS 125552 | OP751053 | OP751055 | OP751051 | OQ630513 | OQ630511 |
| *Torula camporesii* | KUMCC19-0112^T^ | MN507402 | MN507400 | MN507401 | MN507403 | MN507404 |
| *Torula canangae* | MFLUCC 21-0169^T^ | OL830816 | OL966950 | – | ON032379 | – |
| *Torula chiangmaiensis* | KUMCC16-0039^T^ | KY197856 | MN061342 | KY197863 | KY197876 | – |
| *Torula chinensis* | UESTCC 22.0085^T^ | OQ128004 | OQ127986 | OQ127995 | – | – |
| *Torula chromolaenae* | KUMCC16-0036^T^ | KY197860 | MN061345 | KY197867 | KY197880 | KY197873 |
| *Torula fici* | CBS 595.96^T^ | KF443385 | KF443408 | KF443387 | KF443402 | KF443395 |
| *Torula fici* | KUMCC16-0038 | KY197859 | MN061341 | KY197866 | KY197879 | KY197872 |
| *Torula gaodangensis* | MFLUCC17-0234^T^ | NG_059827 | MF034135 | NG_063641 | – | – |
| *Torula goaensis* | MTCC 12620^T^ | NG_060016 | NR_159045 | – | – | – |
| *Torula herbarum* | CPC24414^T^ | KR873288 | KR873260 | – | – | – |
| *Torula hollandica* | CBS 220.69^T^ | NG_064274 | NR_132893 | KF443389 | KF443401 | KF443393 |
| *Torula hydei* | KUMCC16-0037^T^ | MH253926 | MN061346 | MH253928 | MH253930 |  |
| *Torula lancangjiangensis* | HKAS 112709^T^ | NG_081516 | NR_175706 | NG_078759 | MW729785 | MW729780 |
| *Torula longan* | ZHKUCC 22-0121^T^ | OR194027 | OR194035 | OR194032 | OR228537 | OR228535 |
| *Torula luguhuensis* | KUNCC 22-12528 | OQ947766 | OQ729758 | – | OQ999004 | OQ999002 |
| *Torula mackenziei* | MFLUCC 13-0839^T^ | KY197861 | MN061344 | KY197868 | KY197881 | KY197874 |
| *Torula mackenziei* | HKAS 112705 | MW879525 | MW723058 | MW774581 | – | – |
| *Torula masonii* | CBS 245.57^T^ | NG_058185 | NR_145193 | – | – | – |
| *Torula masonii* | MFLUCC 20-0239 | MW412517 | MW412523 | MW412514 | – | – |
| *Torula phytolaccae* | ZHKUCC 22-0107^T^ | ON611800 | ON611796 | ON611798 | ON660881 | ON660879 |
| *Torula pluriseptata* | MFLUCC 14-0437^T^ | KY197855 | MN061338 | KY197862 | KY197875 | KY197869 |
| *Torula polyseptata* | MFLUCC:17-1495 | MT214476 | MT214382 | MT214427 | MT235791 | MT235830 |
| *Torula sichuanensis* | UESTCC 22.0087^T^ | OQ127999 | OQ127981 | OQ127990 | - | - |
| *Torula suae* | KUNCC 22-12433 | NG_243740 | NR_198282 | NG_242856 | OP471618 | - |
| *Torula submersa* | UESTCC 22.0086^T^ | OQ128003 | OQ127985 | OQ127994 | OQ158978 | OQ158968 |
| *Torula longiconidiophora* | UESTCC 22.0125 | OQ128002 | OQ127984 | OQ127993 | OQ158976 | OQ158972 |
| *Torula longiconidiophora* | HLG 0728821 | OQ128001 | – | OQ127992 | OQ158977 | OQ158967 |
| *Torula longiconidiophora* | HLG 0728952 | OQ128002 | – | OQ127993 | OQ158976 | OQ158972 |
| *Torula* *sundara* | HKAS 124486 | OR470713 | OR470708 | OR470703 | OR753786 | OR753781 |
| *Torula* *sundara* | HKAS 124487 | OR470714 | OR470709 | OR470704 | OR753787 | OR753782 |
| *Torula* *sundara* | MFLU 21-0089 | OM287866 | OM276824 | – | – | – |
| *Torula thailandica* | MFLU:19-2856 | MN907428 | MN907426 | MN907427 | – | – |
| *Trematosphaeria grisea* | CBS 332.50 ^T^ | NG_057979 | NR_132039 | NG_062930 | KF015698 | KF015720 |
| *Trematosphaeria pertusa* | CBS 122368 ^T^ | NG_057809 | NR_132040 | FJ201991 | KF015701 | FJ795476 |
| *Tubeufia abundata* | MFLUCC 17-2024 ^T^ | MH558894 | MH558769 | – | MH550961 | MH551095 |
| *Tubeufia aquatica* | MFLUCC 16-1249 ^T^ | KY320539 | KY320522 | – | KY320556 | MH551142 |
| *Tubeufia hainanensis* | GZCC 22-2015 ^T^ | OR030835 | OR030842 | – | OR046679 | OR046685 |
| *Tubeufia hainanensis* | GZCC 23-0589 | OR066421 | OR066414 | – | OR058860 | OR058853 |
| *Tzeanania taiwanensis* | NTUCC 17-005 ^T^ | MH461120 | MH461123 | MH461126 | MH461130 | MH461128 |
| *Tzeanania taiwanensis* | NTUCC 17-006 | MH461121 | MH461124 | MH461127 | MH461131 | MH461129 |
| *Wicklowia aquatica* | CBS 125634 ^T^ | MH875044 | OM322822 | GU266232 | – | GU371813 |
| *Wicklowia fusiformispora* | N-KR1 ^T^ | MZ538567 | MZ538533 | MZ538576 | MZ567108 | – |
| *Xenomassariosphaeria clematidis* | MFLUCC 14-0923 ^T^ | MT214571 | MT310616 | – | MT394630 | – |
| *Xenomassariosphaeria rosae* | MFLUCC 15-0179 ^T^ | MG829092 | – | MG829192 | – | – |
| *Xenopyrenochaetopsis pratorum* | CBS 445.81 ^T^ | GU238136 | MH861363 | NG_062792 | – | KT389671 |
| *Xiuguozhangia broussonetiae* | MFLUCC 24-0258 ^T^ | PQ137419 | PQ137417 | PQ137421 | PQ488461 | PQ488459 |
| *Xiuguozhangia broussonetiae* | MFLUCC 24-0259 | PQ137420 | PQ137418 | PQ137422 | – | PQ488460 |
| *Zopfia rosatii* | CBS 427.62 ^T^ | NG_066246 | NR_160090 | – | – | – |

FIGURE S1. Maximum likelihood (ML) phylogenetic tree inferred from an IQ-TREE analysis of representative species in 93 families of *Pleosporales* based on combined LSU, ITS, SSU, *tef1-α*, and *rpb2* sequence data. Branch support values are shown at the nodes in the order: ML bootstrap support (ML-BS) / gene concordance factor (gCF) / site concordance factor (sCF). ML bootstrap values were calculated from 1,000 non-parametric replicates. The tree is rooted with members of *Tubeufiales*.
