# Supplementary material for: Phenotypic plasticity and cryptic generic enigma in Pleosporales: Establishing Sporidesmioidesaceae fam. nov. and Rostriconidium synnematicum sp. nov. from India
Source: MycoKeys. 2026 Jul 30;138:1–33. doi: 10.3897/mycokeys.138.184414 (PMC13448397; doi:10.3897/mycokeys.138.184414)

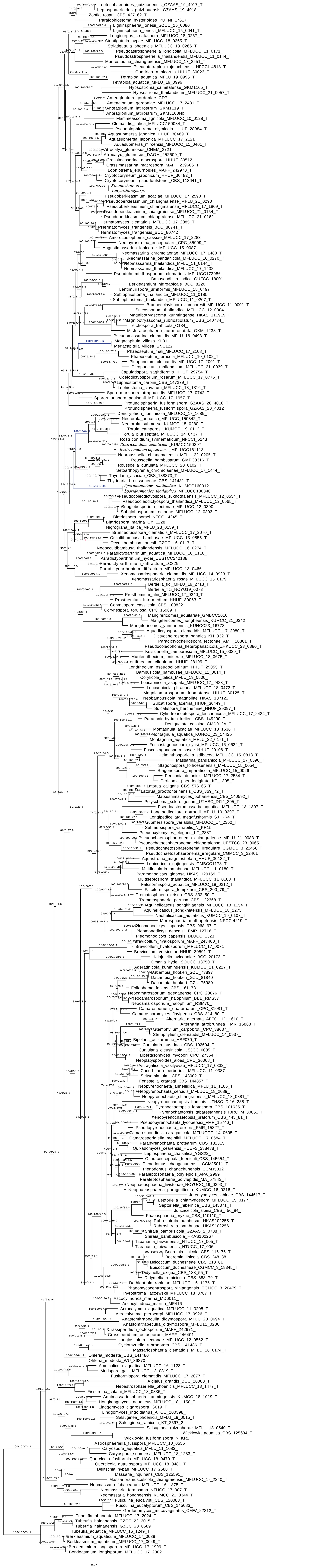

Supplement: Supplementary file 1 [file mycokeys-138-001-s001.pdf]
